# Identification of determinants that allow maintenance of high-level fluoroquinolone resistance in *Acinetobacter baumannii*

**DOI:** 10.1128/mbio.03221-24

**Published:** 2024-11-26

**Authors:** Efrat Hamami, Wenwen Huo, Juan Hernandez-Bird, Arnold Castaneda, Jinna Bai, Sapna Syal, Juan C. Ortiz-Marquez, Tim van Opijnen, Edward Geisinger, Ralph R. Isberg

**Affiliations:** 1 Department of Molecular Biology and Microbiology, Tufts University School of Medicine, Boston, Massachusetts, USA; 2 Department of Biology, Tufts University, Medford, Massachusetts, USA; 3 Department of Biology, Northeastern University, Boston, Massachusetts, USA; 4 Department of Biology, Boston College, Chestnut Hill, Massachusetts, USA; 5 Innovation Laboratory, Broad Institute, Cambridge, Massachusetts, USA; University of Washington, Seattle, Washington, USA

**Keywords:** *Acinetobacter*, efflux pumps, drug resistance mechanisms

## Abstract

*Acinetobacter baumannii* is associated with multidrug-resistant infections in healthcare settings, with fluoroquinolones such as ciprofloxacin being currently ineffective. Clinical isolates largely harbor mutations in the GyrA and TopoIV fluoroquinolone targets, as well as mutations that increase expression of drug resistance-nodulation-division (RND) efflux pumps. Factors critical for maintaining fitness levels of pump overproducers are uncharacterized despite their prevalence in clinical isolates. We, here, identify proteins that contribute to the fitness of fluoroquinolone-resistant (FQR) strains overexpressing three known RND systems using high-density insertion mutagenesis. Overexpression of the AdeFGH efflux pump caused hypersensitization to defects in outer membrane homeostatic regulation, including lesions that reduced lipooligosaccharide (LOS) biosynthesis and blocked production of the major *A. baumannii* porin. In contrast, AdeAB pump hyperexpression, in the absence of elevated *adeC* expression (the outer membrane component of the pump), was relatively tolerant to loss of these functions, consistent with the outer membrane protein being the primary disruptive component. Surprisingly, overexpression of proton-transporting efflux pumps had little impact on cytosolic pH, consistent with a compensatory response to pump activity. The most striking transcriptional changes were associated with AdeFGH pump overexpression, including the activation of the phenylacetate (PAA) degradation regulon. Disruption of the PAA pathway resulted in cytosolic acidification and defective expression of genes involved in protection from oxidative stress. These results indicate that RND efflux pump overproduction is compensated by maintenance of outer membrane integrity in *A. baumannii* to facilitate fitness of FQR isolates.

**IMPORTANCE:**

*Acinetobacter baumannii* is a pathogen that often causes multidrug-resistant infections in healthcare settings, presenting a threat to the efficacy of known therapeutic interventions. Fluoroquinolones such as ciprofloxacin are currently ineffective against a majority of clinical *A. baumannii* isolates, many of which express pumps that remove this antibiotic class from within the bacterium. Three of these pumps can be found in most clinical isolates, with one of the three often hyperproduced at all times. In this study, we identify proteins that are necessary for the fitness of pump hyperproducers. The identified proteins are necessary to stabilize the outer membrane and allow the cytoplasm to tolerate the accumulation of ions as a consequence of excess pump activity. These results point to strategies for developing therapies that combine known antibiotics with drugs that target proteins important for survival of strains hyper-expressing efflux pumps.

## INTRODUCTION

*Acinetobacter baumannii* is a Gram-negative bacterium primarily associated with diseases in healthcare settings (1–3). The microorganism has the potential to cause a diverse range of diseases including pneumonia, wound, and urinary tract infections (1–3). Of great concern is its role in causing ventilator-associated pneumonia, which may be closely tied to its ability to grow in biofilms on indwelling devices (2–6). Particularly challenging is the high incidence of multidrug-resistant (MDR) *A. baumannii* in hospitalized patients, leaving afflicted individuals with few treatment options (1–3). Although there was a reduction in nosocomial cases in the USA from 2013 to 2019, the severe acute respiratory syndrome coronavirus 2(SARS-CoV-2) pandemic saw a large jump in cases that illustrate the difficulties in suppressing diseases by this organism (3). Furthermore, the increasingly common resistance profiles of clinical isolates that are refractory to most antimicrobial therapy regimens indicate that the pathogen poses a continued threat to successful treatment (1–3, 7–10). These clinical isolates include a high proportion that are MDR or have decreased susceptibility to various single agents (1–3, 7–10). This has prompted the World Health Organization to report that *A. baumannii* is a high priority candidate for research and development of new antimicrobials (8). For this reason, there is a high demand for novel antimicrobials or improved combination therapies with which we can combat this pathogen.

There are a variety of means by which *A. baumannii* acquires resistance, including the acquisition of drug resistance elements via horizontal gene transfer (HGT), the selection of mutations that inactivate drug target sites, and hyperexpression of preexisting drug efflux pumps (11–15). Pathogens not only harbor a multitude of efflux systems, some of which are acquired by HGT, but many have broad substrate specificities (11–15), stimulating efforts to discover effective pump inhibitors (16). Unfortunately, there has been no indication of progress in bringing these inhibitors to the clinic, although several have proven effective in interfering with drug action or synergizing with clinically useful antibiotics using laboratory culture conditions or in animal models (16). Nevertheless, given the relentless patterns of resistance acquisition after the use (and misuse) of single-target antibiotics, a treatment regimen that uses a combination of readily available antimicrobials working in synergy could be effective in interfering with resistance evolution. This study approaches this concept by determining candidate proteins that could be targeted together with clinically proven classes of antibiotics.

One drug class that has reduced effectiveness against nosocomial pathogens is the fluoroquinolones (2, 17). Resistance in clinical isolates primarily selects for mutations that disrupt drug binding to the targets topoisomerases II and IV, encoded for by *gyrA* and *parC* genes, respectively (17). In *A. baumannii*, the highest levels of fluoroquinolone resistance (FQR) are found in isolates that have increased expression of drug efflux pumps in addition to mutations in the drug targets. These drug efflux pumps belong to the resistance-nodulation-division (RND) family, in which overexpression is commonly driven by mutations in regulatory elements that result in constitutive expression (18–23). The three major efflux pumps commonly associated with FQR in *A. baumannii* clinical isolates are encoded for by *adeAB*, *adeFGH*, and *adeIJK*, which are controlled by *adeRS* (encoding a two-component system), *adeL* (a LysR type regulator), and *adeN* (a TetR repressor) regulators, respectively (18–23). Among these three, the AdeN-regulated pump is associated with intrinsic resistance to fluoroquinolones, as this is the only RND pump in *A. baumannii* that has measurable levels of expression in the absence of activating mutations (17, 22, 23).

These pump systems traverse both membranes (24–30), and overproduction requires that the envelope accommodates an elevated population of protein complexes not present in drug-sensitive isolates, putting potential stress on the inner and outer membrane. Therefore, the stability and assembly of these proteins as well as the fitness of *A. baumannii* during exponential growth may rely upon bacterial functions that work to maintain cell envelope integrity or prevent intrinsic toxicity of the overexpressed efflux system. Our work focuses on identifying genetic interactions in these high-level FQR strains that allow them to maximize fitness, with the goal of identifying proteins necessary to buffer the deleterious effects of pump hyperexpression that could lead to decreased fitness levels in these strains. Such mutations are predicted to identify targets for drugs that are specifically toxic for pump overproducers.

In this study, we used dense transposon sequencing and transcriptomics strategies to identify proteins necessary to maintain fitness of *A. baumannii* efflux pump-overexpressing strains. We demonstrate that strains harboring overexpressing mutations in *adeN* and *adeS* show surprising robustness and identify targets of vulnerability in over-expressors of the AdeL-regulated pump. These targets are central players in maintaining cell envelope homeostasis. We also connect the expression of the phenylacetate catabolic pathway as a response to pump hyperexpression that leads to maintaining cytosolic pH and maximizing protection from reactive oxygen species.

## RESULTS

### Identification of mutations that depress fitness of fluoroquinolone-resistant strains overexpressing RND efflux pumps

**Fig 1:**
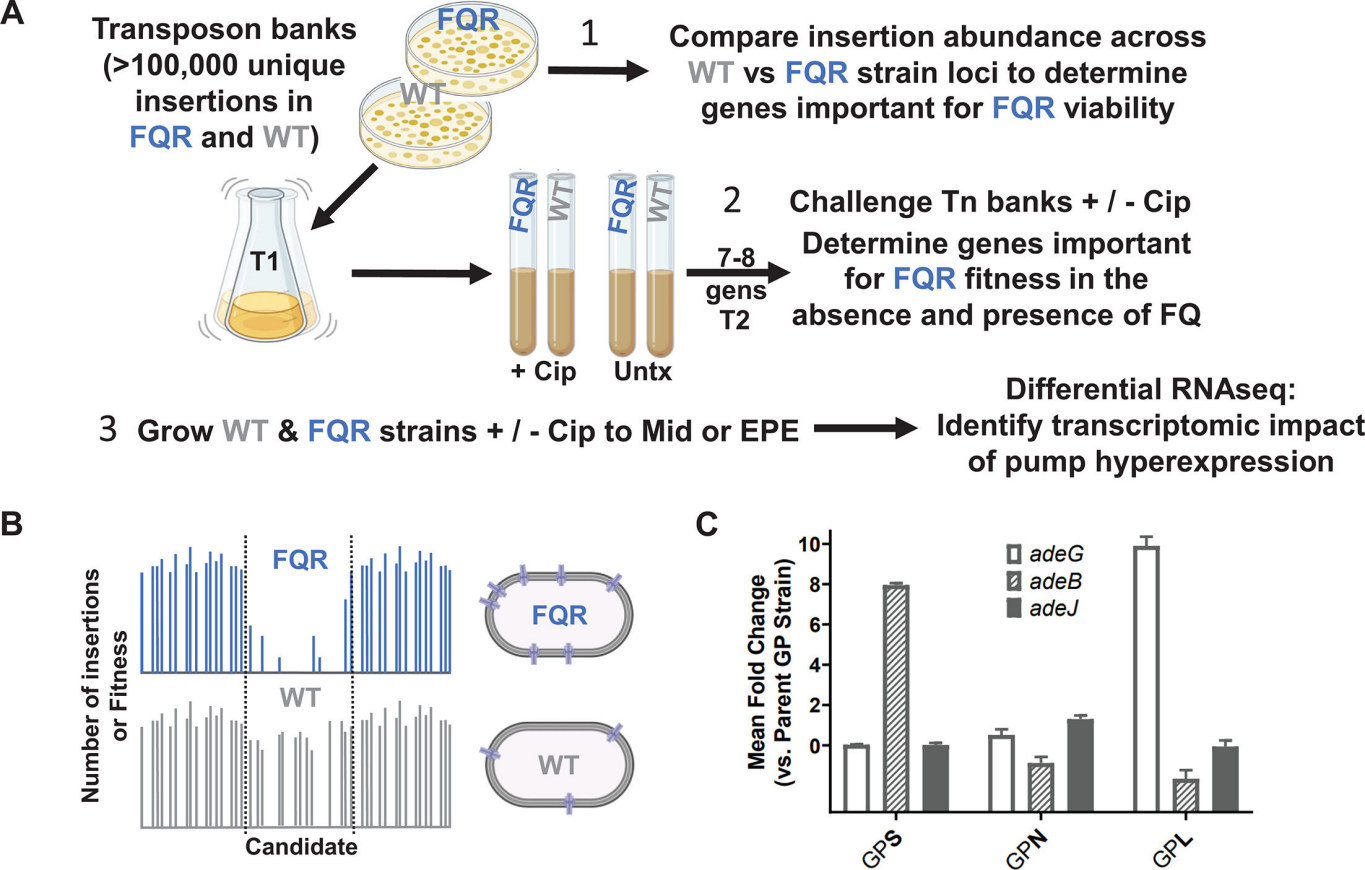
Uncovering the limitations of high-level FQR strains with enhanced RND pump expression. (**A**) Diagram for the three experimental approaches as described in Materials and Methods (created with BioRender.com). (1) Approach #1: transposon insertion libraries were created in FQR strains overexpressing efflux pumps and the drug-susceptible WT strain. Insertion abundance across the genome between the two strain types was compared to identify genes with fewer insertions in the FQR strain background than in WT (see B for example). (2) Approach #2: transposon insertion banks were challenged with sub-MIC levels of CIP, parallel to those grown without drug. Fitness of all transposon mutants was calculated and compared between the strain types. (3) Approach #3: strains were grown in the presence or absence of CIP to exponential or post-exponential phase in the presence or absence of drug and transcriptomic data were obtained to discover differentially regulated pathways. (**B**) Example of insertion abundance in a single gene cluster in two strain backgrounds. (**C**) Activating mutations in efflux pump regulatory mutants specifically activate their cognate pumps. RNA was isolated from the noted strains and relative transcription determined by quantitative reverse transcription PCR (q-rtPCR) (Materials and Methods). Displayed is mean fold change ± SEM of each target as compared to the parental GP strain (Log2 scale). At least three independent biological replicates were used for each strain. FQR, fluoroquinolone resistant; Mid, mid-exponential phase; EPE, early post-exponential phase; Untx, untreated; GPL, *gyrA parC adeL*; GPN, *gyrA parC adeN*; GPS, *gyrA parC adeS*; GP, *gyrA parC*.

To identify genes that are important for maintaining the fitness of strains having high-level FQR, we isolated mutations in the *A. baumannii* ATCC 17978UN strain background that are viable in high concentrations of ciprofloxacin (CIP; Materials and Methods). These strains were then subjected to a mutagenesis strategy (Fig. 1A) that identified insertion mutations that had lowered viability in an FQR strain background compared to the wild type (called strain WT) (Fig. 1B). Three FQR strains were interrogated, each of which harbored *gyrA*(S81L) and *parC*(S84L) mutations that are responsible for fluoroquinolone resistance in most Gram-negative organisms and are commonly found in the clinic (called strain GP). The three strains were distinguished from each other by having distinct mutations in loci that regulate the expression of clinically relevant *A. baumannii* RND drug efflux pumps. The mutation in a component of a two-component regulatory system *adeS*(R152S) upregulates the AdeAB pump, the activating *adeL*(∆I335A336) mutation upregulates the AdeFGH pump, while a knockout in the repressor *adeN*::ISAba1 increases expression of the AdeIJK pump (Fig. 1C). It should be noted that under conditions of AdeAB pump overexpression, AdeK is used as the outer membrane component which is expressed at wild-type levels in the presence of the *adeS*(R152S) pump-activating mutation (Fig. 1C) used in this study (31, 32).

**Fig 2:**
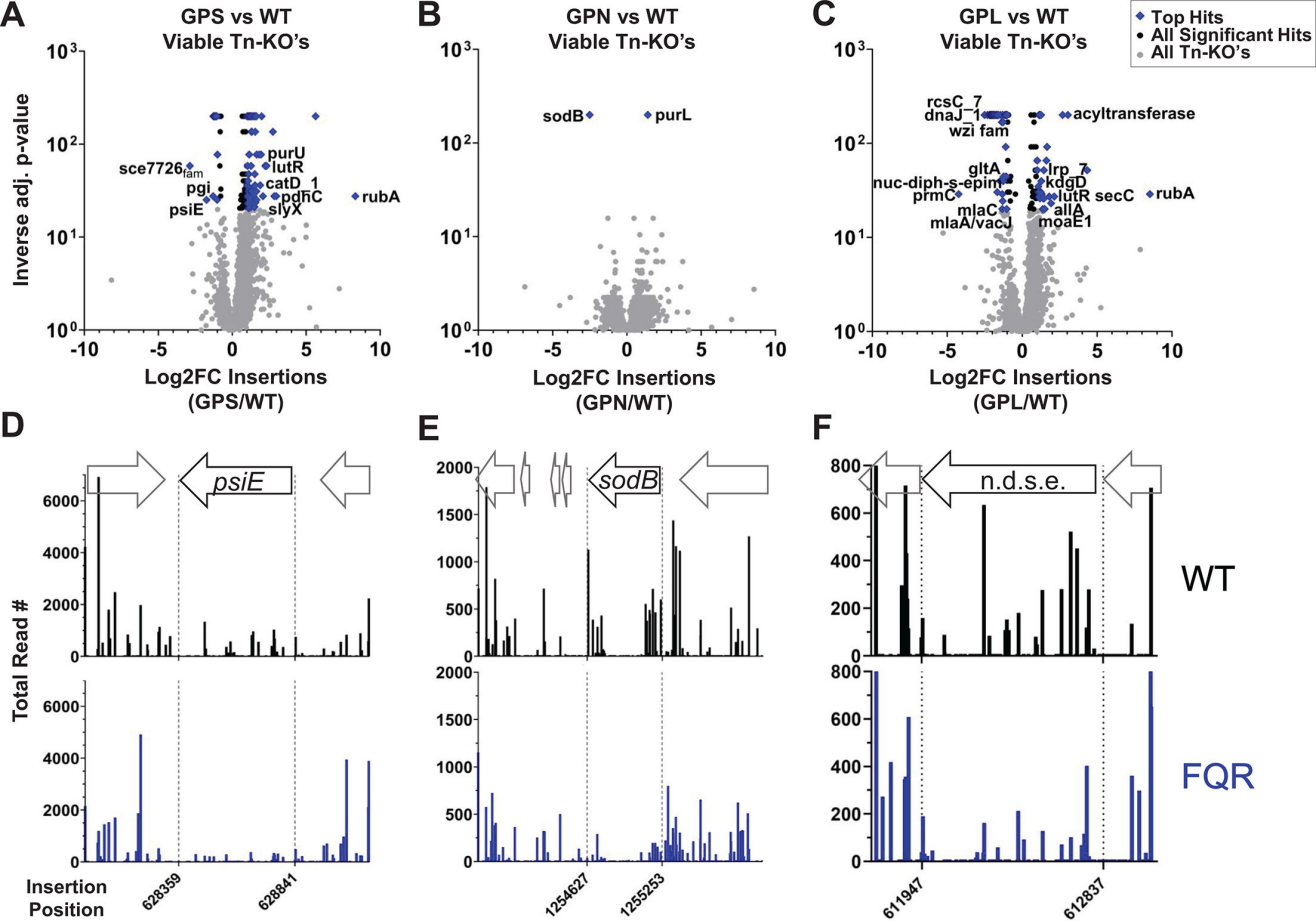
Discovery of genes with lowered tolerance for insertions in the high-level FQR strains. (**A–C**) Volcano plots in which statistical significance is plotted as a function of the differences in insertion abundance between each FQR strain and WT based on TRANSIT algorithm (33). All adjusted inverse *P*-values = 0 were assigned the value of 0.005 to allow graphing. Black and blue: genes with adjusted *P*-values <0.05. Values for all other genes are shown in gray. (**D–F**) Number of reads plotted as a function of insertion site for noted genes as well as flanking regions. FQR, fluoroquinolone resistant; GPL, *gyrA parC adeL*; GPN, *gyrA parC adeN*; GPS, *gyrA parC adeS*.

Transposon (Tn) mutagenesis was used to determine candidates important for viability or fitness in either the absence or presence of drug. Between 110,000 and 150,000 unique transposon mutants were created in all three FQR and the WT strain backgrounds, reaching pool saturation levels of about 37%–50% based on targets predicted for the *himar-1* transposon (Materials and Methods). Each bank was sequenced post-harvest and transposon-proximal sequences were amplified to identify the locations of insertions in each strain background and to determine overall pool saturation. These sequence data were also utilized to identify insertion site distributions that were present in each strain, because insertions in genes predicted to be essential or cause fitness defects in a specific pump overproducer strain are predicted to be underrepresented relative to the WT strain. Per our first approach (Fig. 1A), insertion abundance throughout the genome was compared between strain backgrounds using the TRANSIT resampling method to identify genes that have fewer insertions relative to WT in the absence of drug (33) (Fig. 2; Data Set S1), as candidates for genes that are putative conditionally essential targets. To focus on strain-dependent conditional essentiality, 445 genes characterized as essential by TRANSIT’s Gumbel method (34, 35) were excluded from this and downstream analyses using transposon mutagenesis. Data were displayed by the statistical significance as a function of the insertion abundance in each of the pump overproducer strains relative to WT (Fig. 2A through C). Among genes identified in this fashion (passing significance criteria adj. *P* < 0.05) in the absence of fluoroquinolones were *psiE* (*adeS* background), *sodB* (*adeN* background), and “nuc-diph-s-epim” or “n.d.s.e” (*adeL* background), respectively, encoding a phosphate starvation-inducible locus, superoxide dismutase, and a nucleoside diphosphate sugar epimerase more recently implicated in LOS assembly (36) (Fig. 2D through F).

Prior to the exclusion of essential genes, this analysis revealed that *cyoAB* encoding components of the essential ubiquinol oxidase complex harbored significantly fewer insertions in *gyrA parC adeL* (GPL) than in WT (Fig. S1A and B). Its function during aerobic respiration as a generator of the proton motive force may play a role in allowing the bacterium to tolerate proton influx resulting from the drug-proton antiport activity of the RND efflux pump (37, 38). To provide support that lowered Cyo activity reduces fitness in the presence of *adeL*∆I335A336, we used CRISPR interference (CRISPRi) to knock down expression of the *cyoABCDE* operon in the GPL and WT strains and observed growth in broth culture. To this end, an anhydrotetracycline (aTc)-inducible dCas9 was inserted at the *att*Tn7 locus in both strains and transformed with a plasmid harboring a constitutive single-guide RNA (sgRNA) (Materials and Methods). As a control, the non-targeting original vector (labeled NC-sgRNA; Table S1) was used. Growth was monitored in cultures grown for 5.5 or 6 hours after dilution to low density in lysogeny broth (LB) and aTc. We saw a fitness defect when expression of the ubiquinol oxidase system was knocked down in both the WT and the GPL strains, with the defect enhanced in WT treated with 100 ng/mL aTc (Fig. S1C). Quantification of *cyoABCDE* operon transcript levels after aTc addition showed that expression was silenced in both strains at comparable efficiency compared to controls (Fig. S1D).

Notably, there were very few insertions in both the WT and GPL strains which is an indication of gene essentiality. As a consequence, although the locus was distinguished among those showing the most highly significant difference in insertion efficiencies (Fig. S1B), these differences were measured by degrees of essentiality (Fig. S1E). Furthermore, the average number of viable insertion mutants per *himar-1* transposon location (TA) among the cumulative WT pools was approximately 258 and in GPL about 440. While the loci highlighted from these analyses demonstrate significantly different insertion efficiencies, these differences were likely artifacts of plating efficiency rather than conditional essentiality. Therefore, a second strategy was pursued to measure fitness defects during growth in broth culture to attempt to identify more clearcut differences between the two strain backgrounds.

### The FQR mutants have increased vulnerability to disruption of cell envelope homeostasis

**Fig 3:**
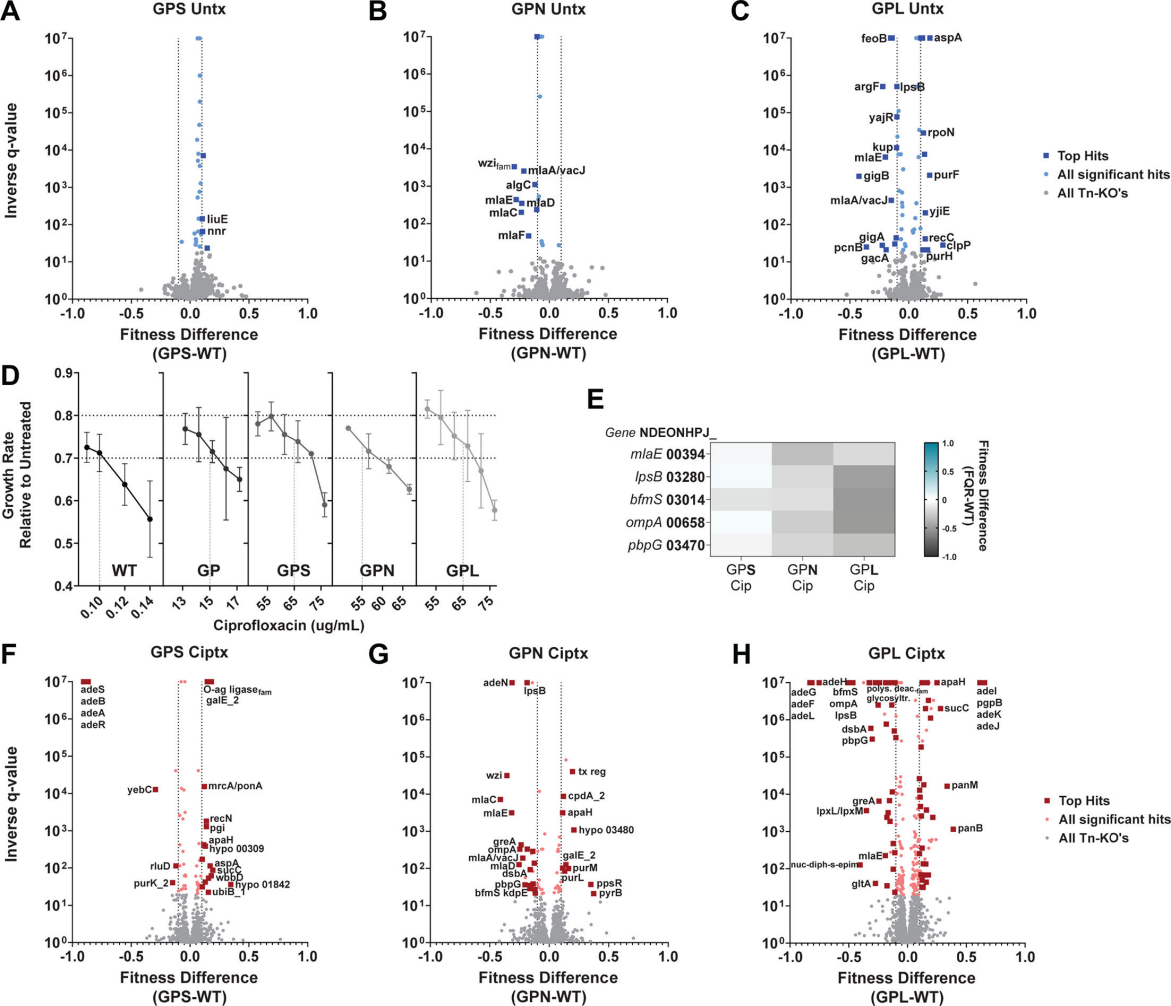
Maintenance of cell envelope homeostasis supports the fitness of efflux pump overproducer strains. (**A–C, F–H**) Statistical significance plotted as a function of relative fitness comparing identical mutations in FQR and WT strains grown in the absence (**A–C**) or presence (**F–H**) of sub-MIC levels of CIP. (**D**) Growth rates at noted CIP concentrations to identify conditions allowing 20%–30% growth inhibition in the FQR and WT strains. (**A–C**) Blue or (**F–H**) red: mutations above the false discovery rate (FDR) = 5%, q < 0.05 as determined by Benjamini and Hochberg (39). Notable findings are labeled. All q-values <0.000001 assigned the value of 0.000001 for graphing purposes. (**E**) Heatmap showing fitness of insertions in each gene in noted strain backgrounds. Selected genes with Tn disruptions yielding lowered fitness in the FQR strains are shown in the presence of CIP. Gene names are followed by accession numbers which are preceded by “NDEONHPJ_” per the ATCC 17978 strain annotations. These data exclude genes characterized as essential and genes pertaining to prophages or plasmids. GP, *gyrA parC.*

To attack this problem using a second approach, multiple small Tn insertion pools (~8–16k unique insertions) were constructed in each background, and each was grown for seven to eight generations in the absence of antibiotics to determine the relative fitness levels of individual gene disruptions (>79,000 unique insertions in total for each background; Materials and Methods). The difference in fitness levels between an individual pump overproducer and the wild type was then determined, and statistical significance was plotted as a function of fitness differences in the two backgrounds (Fig. 3A through C). In the absence of drug, the *gyrA parC adeS* (GPS) mutant was particularly tolerant of insertion mutations relative to the WT control (Fig. 3A; Data Set S2). For the *gyrA parC adeN* (GPN) and GPL strains in the absence of drug, among the proteins identified are those affecting membrane integrity including those that modulate lipid trafficking and LOS synthesis (Fig. 3B and C; Data Set S2). Notably, the GPS strain which shows no upregulation of the outer membrane component of the RND systems was the most tolerant of disruption in these pathways (Fig. 3A).

To determine if antibiotic treatment increased the spectrum of mutations that sensitize pump overproducers, each of the strains was treated at various CIP concentrations to identify conditions that resulted in 20%–30% growth rate inhibition (Fig. 3D). Using drug concentrations that reproduced these growth rates, each of the insertion banks was grown seven to eight generations in the presence of CIP (17), cultures were harvested, gDNA was isolated, insertion sites were selectively amplified and sequenced, and the abundance of each insertion was determined using the MAGenTA pipeline (17, 40, 41) (Materials and Methods). From these data sets, the fitness scores of gene disruptions were normalized to a LOWESS curve to avoid the position bias exhibited with CIP treatment (Fig. S2). The differences in fitness levels between an individual pump over-expressor and the wild type were then used to identify mutants that preferentially hypersensitize the pump-overexpressing strain to CIP (Fig. 3E through H; Data Set S2).

In general, treatment with drug increased the number of insertion mutations that resulted in fitness defects in the pump hyper expressing strains compared to WT (compare Fig. 3A through C and E through H). Primary targets that preferentially sensitize at least two FQR strains during CIP treatment were concentrated in loci related to envelope homeostasis, including *bfmS* which affects a collection of cell envelope processes (42) and *pbpG* which is involved in peptidoglycan degradation (41) (Fig. 3E through H). Even so, the magnitude of defects was consistently higher for mutations in GPL compared to the GPS background, with mutations in the GPN background often showing intermediate effects (Fig. 3E). As in the absence of drug, the GPS strain showed higher tolerance than the GPL and GPN strains for mutations in the same pathways (Fig. 3E through H).

**Fig 4:**
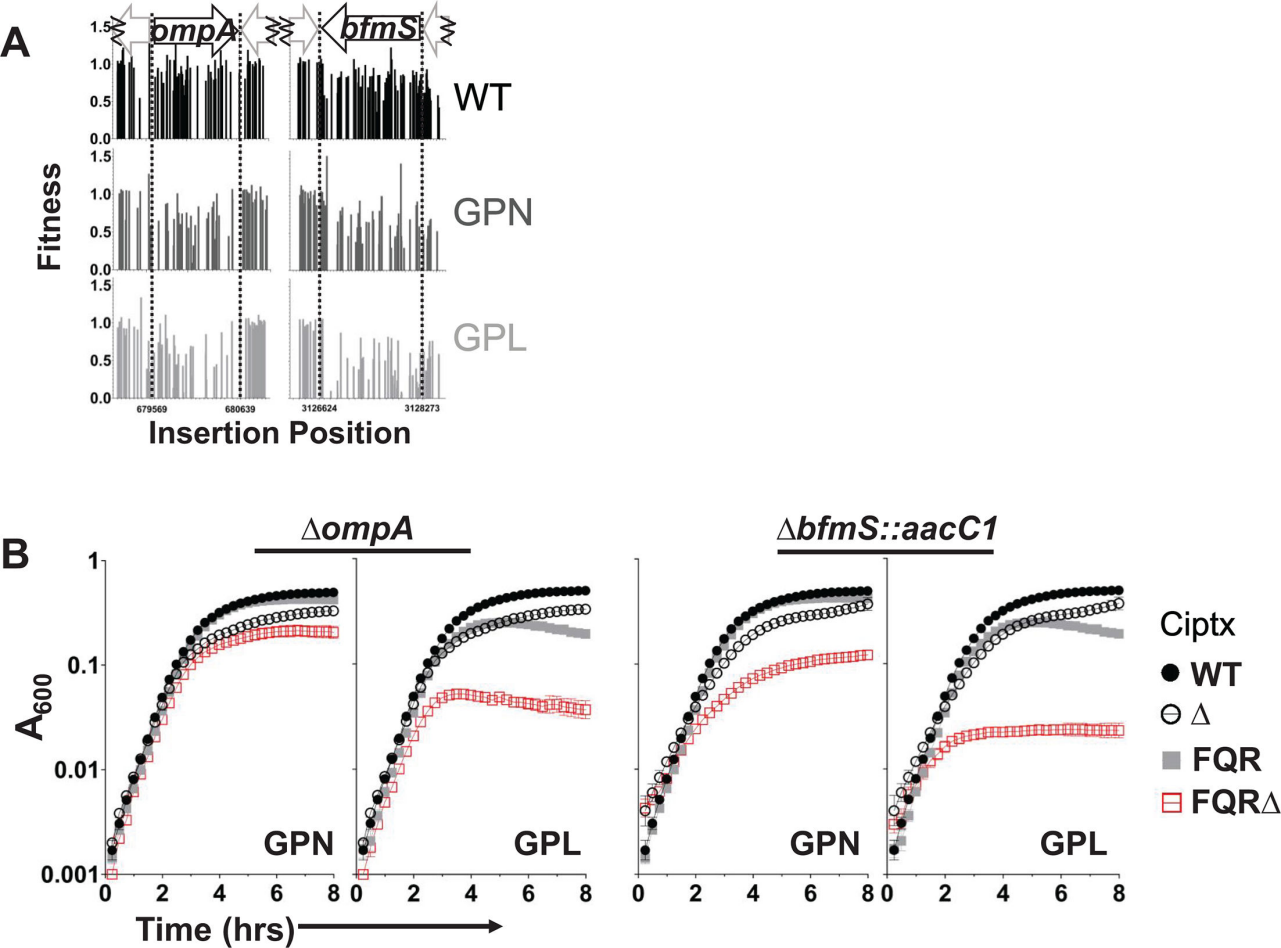
OmpA and BfmS are critical fitness determinants for *adeL*∆I335A336 strains during CIP exposure. (**A**) The Single Fitness pipeline of MAGenTA was used to evaluate the fitness of Tn mutants at each position across the loci shown, post CIP treatment. (**B**) Null mutations in the FQR strain backgrounds grown in the presence of CIP compared to the WT background. Same experimental data for the WT strain and single deletion mutants are shown in each graph for comparative purposes. Growth was monitored by taking absorbance measurements every 15 min in 96-well plates in the presence of CIP (0.1 µg/mL for the WT, 55 µg/mL for the GPN, and 65 µg/mL for the GPL parent strains and deletion mutants in those respective backgrounds). Displayed are means ± SEM (*n* ≥ 3). Of note, GPL∆*ompA* has an additional mutation in *lpxK* (I283V). Ciptx, CIP treated.

**Fig 5:**
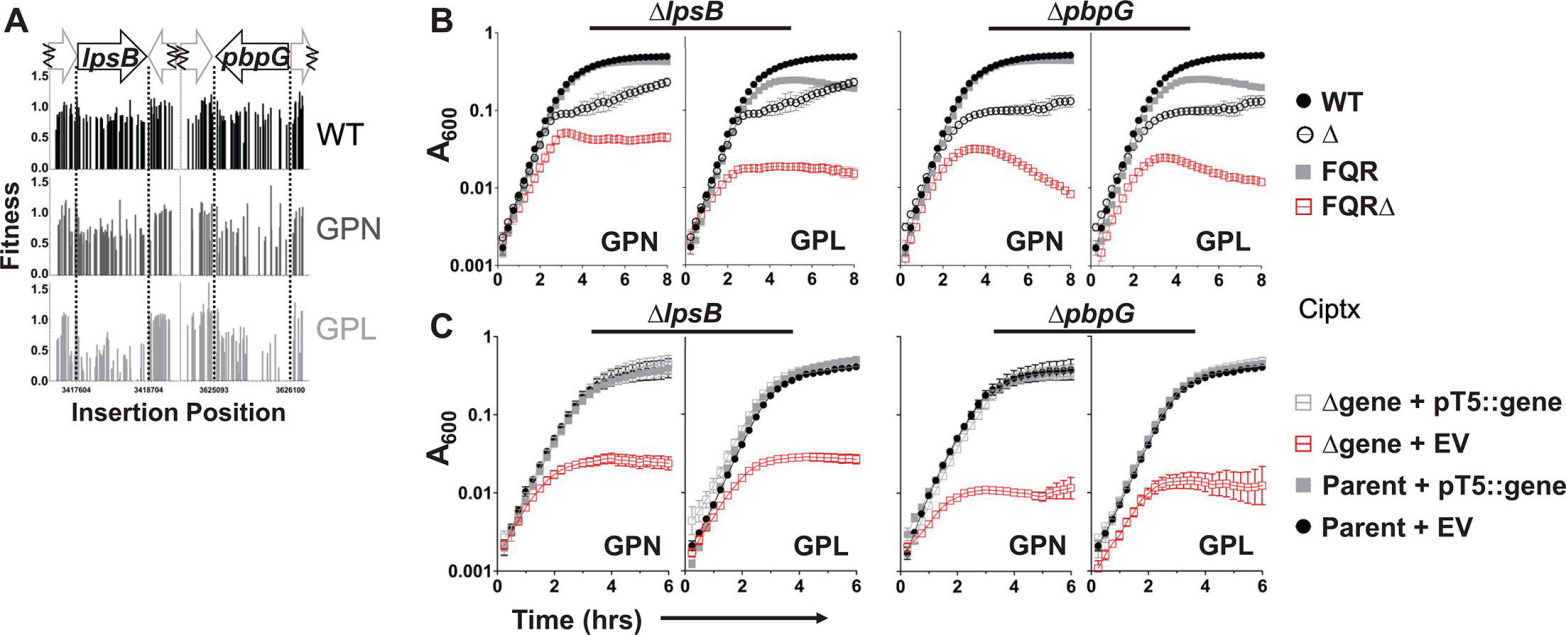
LpsB and PbpG are required for the AdeN (GPN) and AdeL (GPL) mutants for optimal fitness in the presence of CIP. (**A**) Transposon mutant fitness post CIP treatment at each insertion position across the selected loci. (**B**) Growth of the indicated strains in the presence of CIP was monitored as in Fig. 4, displayed as mean ± SEM (*n* ≥ 3). Data for the WT strain and single deletion mutants from identical cultures are shown in each graph for comparative purposes. (**C**) Growth of deletion mutants in LB + 65 µg/mL CIP + 1 mM IPTG to induce expression of the indicated genes complemented *in trans* in GPN (column 2) or GPL (column 4) backgrounds. Data are means ± SEM (*n* ≥ 6). Ciptx, CIP treated; IPTG, isopropyl β-d-1-thiogalactopyranoside; GP, *gyrA parC*; EV, empty vector.

Knockout mutations of candidates were generated as gene deletions in both the FQR and parent strains via homologous recombination to verify their phenotypes (their genotypes confirmed by whole genome sequencing, Materials and Methods). The targets for deletion were chosen based on the following criteria: (i) they resulted in significant fitness defects in at least two of the FQR strains (q ≤ 0.05); and (ii) they had severe fitness defects in at least one FQR strain (fitness defect ≥0.25). Bacterial growth was then evaluated in the presence of CIP (Fig. 4 and 5) at concentrations that yielded comparable growth inhibition across strains (Fig. 3D). Based on Tn-seq analysis (Fig. 3E through H), insertion mutants in *bfmS* and *ompA_Ab_* showed increased CIP sensitivity in GPN and GPL compared to the WT strain background (Fig. 4A). Consistent with these findings, growth defects caused by the deletion mutations were exacerbated in the pump-overexpressing strains after CIP treatment as compared to the WT strain background (Fig. 4B). Of particular interest was the behavior of each of the mutants in the GPL background. Both the ∆*bfmS::aacC1* and ∆*ompA* mutants were highly attenuated for growth in the presence of CIP, with growth plateauing far below that observed for controls (Fig. 4B). For instance, when ∆*bfmS::aacC1* was crossed into the GPL background, growth plateau was observed at about 10× lower culture densities than that observed for the GPL parent and 12–20× lower than the same mutation crossed into WT (Fig. 4B). Similarly, when the ∆*ompA* mutation was crossed into GPL, plateaus occurred at 5–8× lower culture density than the GPL parent and about 8× lower than the same mutation crossed into WT (Fig. 4B).

To ensure the growth inhibition was a result of the pump regulator mutation, the consequences of crossing the mutations into the lower-level resistance GP strain were analyzed in comparison to GP carrying a pump-activating mutation in each of the pump regulators (Fig. S3). This allowed a direct comparison between strains differing by a single FQR mutation. The increase in drug sensitivity was more pronounced in the FQR pump mutants carrying the Δ*ompA_Ab_* and Δ*bfmS::aacC1* mutations relative to GP with the same lesions (Fig. S3A).

The pump-overexpressing mutations alter regulatory elements that could be modulating the expression of other unidentified genes that cause fitness alterations. To eliminate this possibility, we determined if the fitness defect of the GPL strain harboring the Δ*ompA_Ab_* and Δ*bfmS::aacC1* mutations was reversed by deleting the *adeFGH* efflux pump genes. Deletion of *adeFGH* reversed the fitness defects caused by the Δ*ompA_Ab_* and Δ*bfmS::aacC1* mutations, consistent with pump overproduction causing the observed fitness defects (Fig. S4F and S5F, respectively). Furthermore, for each mutation tested, the GP strain background showed comparable fitness to the GPLΔ*adeFGH* strain background (Fig. S4H and S5G).

The GPLΔ*ompA_Ab_* strain (Fig. 4B) was found by whole genome sequencing (WGS) to harbor an additional mutation in *lpxK* (I283V), so we created additional strains harboring Δ*ompA_Ab_* via homologous recombination in the GPL background (Materials and Methods) and tested their phenotypes relative to that of GP. The heightened sensitivity of GPL to CIP after introduction of the Δ*ompA_Ab_* lesion was retained (Fig. S4B through D), indicating that the fitness defect was due to the loss of OmpA. Similarly, the GPΔ*bfmS::aacC1* strain in Fig. S3A was found to have an additional mutation upstream of the *bfmR* gene. This mutation dampened the expression of *bfmR* (Fig. S5H; strain #272 GPΔ*bfmS::aacC1* [Fig. S3A] compared to strains #271 GPΔ*bfmS::aacC1* [Fig. S5B] and #304 Δ*bfmS::aacC1* [Fig. 4B]). Additional clones cured of this *bfmR* promotor mutation were created in the GP strain background, and knockout of *bfmS* reproducibly showed minimal fitness alterations of the GP parent in the presence of CIP, while still sensitizing the *adeL* variant that overexpresses the efflux pump (Fig. S5B through E).

Given the strong defects observed with these two mutants, other candidate mutations predicted to interfere with envelope biogenesis were interrogated, including *lpsB* and *pbpG*, which showed lowered insertion mutation fitness in the pump hyper-expressors when compared to WT, particularly in the GPL strain background (Fig. 5A). Deletion mutations were constructed in WT, GP, GPN, and GPL backgrounds, and growth of the eight mutant strains was monitored in the presence of CIP. Loss of PbpG from either the GPN or GPL strain background was particularly detrimental to growth in the presence of low CIP concentrations (Fig. 5). In both GPN and GPL backgrounds harboring ∆*pbpG*, strains initiated growth but culture densities stopped increasing after about 3 hours (Fig. 5B; Fig. S3B). In contrast, the parental GP mutant harboring ∆*pbpG* did not show this phenotype (Fig. S3B). In the case of the ∆*lpsB* mutant, growth plateaued at lower levels when the deletion was analyzed in the GPL mutant (8× lower than parent GPL) compared to GPN (5–7× lower than GPN), consistent with the *adeL*(∆I335A336) mutation being particularly susceptible to envelope stress (Fig. 5B).

To ensure that this phenotype was due to these null mutations, both deletions were complemented *in trans*. The presence of plasmids harboring these genes restored the fitness of both pump overexpression strains, with growth in the presence of CIP indistinguishable from the appropriate parents harboring empty vector (Fig. 5C). WGS of these strains uncovered an *mrcB* (G539C) mutation in the GPN∆*lpsB* background. Complementation of the GPN∆*lpsB* strain, however, restored growth to levels of the parental strain, indicating that the growth defect was caused by loss of *lpsB* and not due to the secondary missense mutation (Fig. 5C). Furthermore, when a deletion of the *adeFGH* efflux pump operon was introduced into the GPL strain harboring either the ∆*lpsB* or the ∆*pbpG* mutations, both growth defects were alleviated, indicating that defective growth was linked to the overproduction of the efflux pump (Fig. S6 and S7).

The absence of PbpG or LpsB has been associated with alterations in *A. baumannii* LOS homeostasis (36, 41). Therefore, LOS profiles of strains disrupted in *pbpG* and *lpsB* were determined by fractionation on SDS-polyacrylamide gels (Fig. S8; Materials and Methods). No significant differences in LOS profiles were detected between the WT or FQR strain backgrounds (Fig. S8A). For the various strains harboring the ∆*pbpG* mutant, there were decreased levels of a species unrelated to LOS, but otherwise the migration and relative concentration of LOS species appeared unaffected (Fig. S8B). In contrast, as expected, ∆*lpsB* mutations resulted in truncated LOS, with similar profiles in each of the efflux pump backgrounds (Fig. S8C). Therefore, LOS is altered by these mutations as previously described, and these alterations were independent of the strain background tested (41).

As reduced A_600_ yields could be indicative of morphological differences between mutants, the various strains were imaged. As expected, the *bfmS::aacC1* and ∆*pbpG* mutants showed morphological alterations that have been previously documented, ranging from stubbier to slightly rounded and enlarged, dependent on the mutation (42, 43). The latter was observed with ∆*lpsB* as well. These alterations were independent of pump overproduction (Fig. S9). In contrast, the ∆*ompA* mutation resulted in no morphological changes (Fig. S9). In response to CIP, the parental strain elongated as anticipated (44, 45), while the mutations that showed morphological changes continued to appear different from their parent strain, with changes ranging from reduced length to wider cells (Fig. S10). While structural deformation from loss of PbpG appears slightly more severe in the pump-overexpressing strain background (Fig. S11), the specific morphological alterations caused by the mutants were observed both in the presence and absence of pump hyperexpression (Fig. S11 to S14). Critically, what differentiated the morphotypes of these lesions between the pump-overexpressing strain and parent strain is that cells in the pump-overexpressing strain background consistently displayed larger volumes (Fig. S11 to S14). We conclude that although the mutations that resulted in enhanced CIP sensitivity in pump overproducer strains resulted in altered morphologies, it is unlikely these changes in morphology explain for the reduced culture densities observed in growth curves of strains that overproduce efflux pumps (Fig. 4 and 5).

### Maintenance of pH homeostasis in the presence of pump overproduction

**Fig 6:**
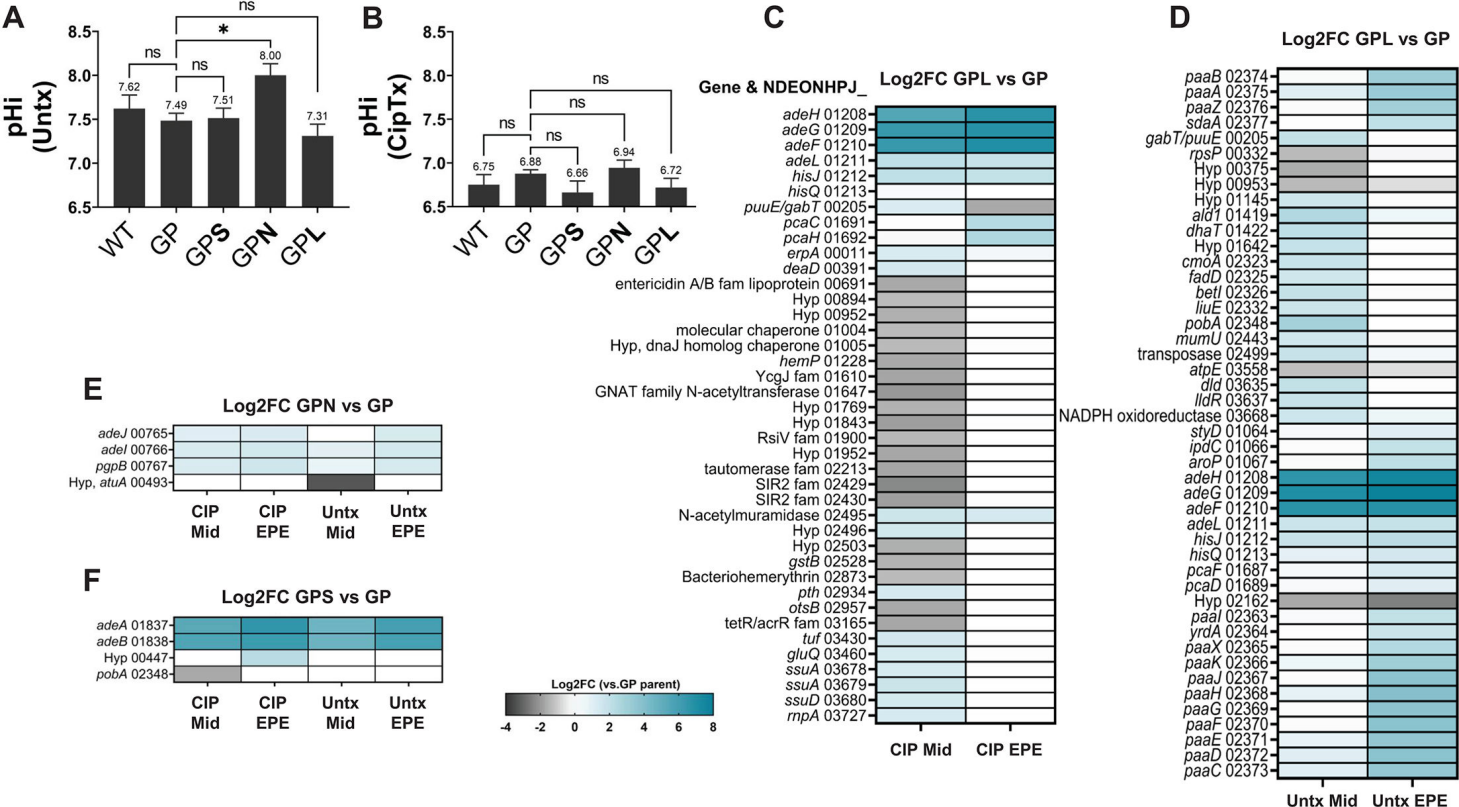
Transcriptional and physiological response to pump overproduction. (A, B) Alterations in intracellular pH (pHi) in response to pump overproduction. Noted strains grown to mid-exponential phase in the absence (**A**) or presence (**B**) of CIP. The pHi was measured using 20 µM BCECF-AM as described (Materials and Methods). Displayed are the means ± SEM for each strain from *n* = 5–8 biological replicates, with *P*-values derived from one-way ANOVA making multiple comparisons. * = *P* < 0.05 (0.0259); ns, not significant. (**C–F**) Transcriptional alterations in response to pump overproductions are magnified in the presence of *adeL*∆I335A336 mutation. Transcriptomic data (*n* = 3) were analyzed comparing high-level FQR pump hyper-expressing strains and the GP parent strain (Materials and Methods). Differences in transcript levels for each comparison are shown via the heatmaps with GP vs GPL (C, +CIP) and (D, -CIP), GP vs GPN (**E**) and GP vs GPS in (**F**). These data exclude genes pertaining to prophages or plasmids. A gene is listed if a significant change in gene expression was observed in at least one condition within the panel. GP, *gyrA parC*; Mid, mid-exponential phase; EPE, early post-exponential phase.

The RND-class efflux pumps are known to function as drug-proton antiporters (46). With this in mind, we investigated whether FQR pump hyperexpression causes *A. baumannii* cytosolic pH to become more acidic in the presence or absence of CIP substrate. WT, the high-level FQR strains, and the GP precursor strains were grown to mid-exponential phase and loaded with BCECF-AM dye to determine intracellular pH (pHi). The pHi values were then determined from a standard curve of cells incubated in pH-adjusted buffers in the presence of carbonyl cyanide 3-chlorophenylhydrazone (CCCP) to equilibrate external and intracellular pH (Materials and Methods). In the absence of antibiotic, the cytosol of GPN, the AdeIJK pump over-expressor, was significantly more basic than the GP parent strain (Fig. 6A), while the cytosolic pH values of the other pump overproducers were otherwise comparable to GP or WT controls (Fig. 6A; one-way analysis of variance [ANOVA], multiple comparisons between all strains and GP, * where *P* < 0.05). To determine if the presence of CIP, which should result in proton-CIP antiport, affects intracellular pH, the experiment was repeated after exposure to CIP at concentrations yielding comparable levels of growth inhibition across strains. CIP treatment lowered the pHi compared to the untreated conditions for all strains. The cytosols of the GPS and GPL strains were fractionally more acidic than the parental GP strain after CIP addition, but the differences did not rise to the level of statistical significance (Fig. 6B; same tests as in Fig. 6A). Imaging shows these strains exhibit similar cell morphologies both in the absence (Fig. S15) and presence of CIP (Fig. S16). These data indicate there may be homeostatic mechanisms to neutralize the effects of pump overexpression that act at either transcriptional or post-transcriptional levels, so the global transcriptional effects of pump overexpression were analyzed in the presence and absence of CIP (Fig. 1A, third approach).

### Large transcriptional changes in response to pump overproduction are limited to the AdeL-regulated pump

To determine if pump overproduction results in transcriptional changes that support fitness, the FQR, low-level resistance GP, and WT strains were cultured in the absence or presence of CIP at concentrations causing 20%–30% growth inhibition and analyzed by RNAseq (Materials and Methods). Samples were collected during exponential phase (A_600_ ~0.5) as well as early post-exponential phase (A_600_ ~1), and differentially regulated genes were identified by comparing the high-level FQR strains and the GP parent strain (Fig. 6C through F; GP vs GPL [C, +CIP] and [D, -CIP], GP vs GPN [E], and GP vs GPS [F], Data Sets S3 and S4). In either the presence or absence of CIP, the GPL strain had the greatest number of differentially expressed genes relative to the control GP strain when compared to the other pump-overexpressing strains (Fig. 6C through F). In fact, hyperexpression of either the AdeS- or AdeN-regulated pumps only altered the gene expression patterns of very few genes in the presence or absence of CIP (Fig. 6E and F). This result echoes the Tn-seq analysis of the three overproducer strains, as the *adeL* mutant had the largest set of mutations that lowered fitness relative to WT (Fig. 2 and 3; Fig. S17 to S19).

**Fig 7:**
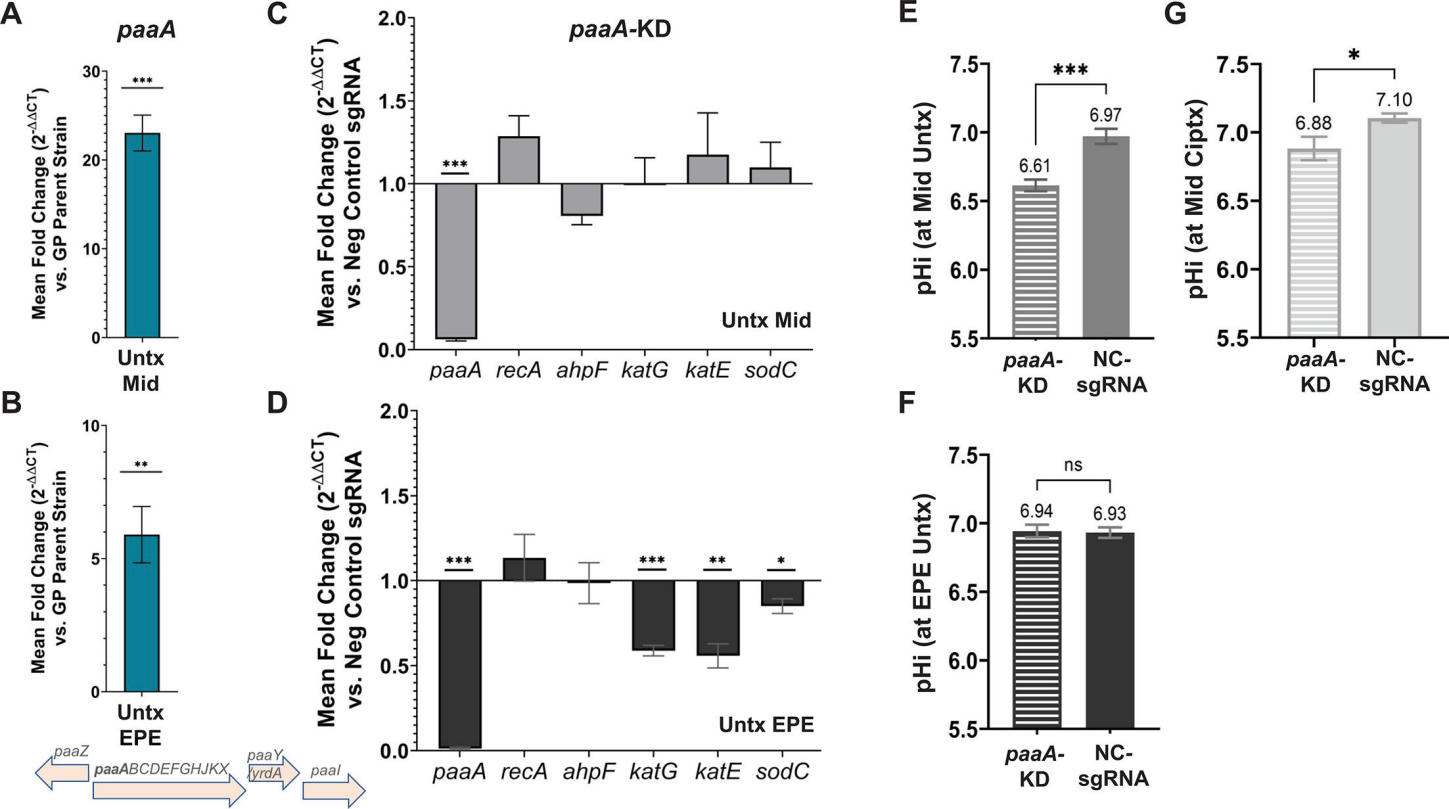
The presence of *adeL*∆I335A336 in GPL results in overexpression of the *paa* operon. RNA was isolated from GP and GPL strains and transcription of *paaA* gene was quantified by q-rtPCR. Strains grown to exponential (**A**) or early post-exponential phase (**B**) in the absence of drug. Data are mean fold change in transcript levels (2^-ΔΔCT^) ± SEM of each target (*n* = 3) compared to parental GP strain, analyzed by two-tailed *t*-test. Significance marked by asterisks; **, *P* = 0.0099; ***, *P* = 0.0004. (**C, D**) Alterations in transcript levels in CRISPRi-depleted *paaA* strain, with knockdown verified as described (Materials and Methods). RNA was prepared from cultures grown to mid-exponential (**C**) or early post-exponential phase (**D**). Data are transcript levels of each target relative to non-targeting negative control (2^-ΔΔCT^) ± SEM (*n* = 3), analyzed by multiple unpaired *t*-test analyses following the two-stage step-up Benjamini, Krieger, and Yekutieli, false discovery rate of 5.00% (47). Significance: *, *P* = 0.020–0.034; **, *P* = 0.002–0.003; ****, *P* < 0.001. (**E–G**). Acidification of cytosol as a consequence of *paaA* depletion. The pHi determined during mid-exponential phase in the absence (**E, F**) or presence (**G**) of CIP. (**F**) Intracellular pH in early post-exponential phase. Data are mean ± SEM from *n* = 5. *P*-values were derived from an unpaired *t*-test comparing the mean pHi values between the two strains. (***, *P* = 0.0009 in E; *, *P* = 0.0420 in G and ns, *P* = 0.8479 in F). Untx, untreated; Ciptx, CIP treated at 45 µg/mL; Mid, grown to mid-exponential phase; EPE, grown to early post-exponential phase; GP, *gyrA parC*.

Among the various transcriptional changes resulting from AdeL-regulated pump hyperexpression, the most notable was upregulation of the *paa* operon, which encodes proteins involved in degradation of phenylacetate (Fig. 6C and D). In the absence of drug, 14 of 46 genes that were differentially regulated in the two backgrounds were from the *paa* regulon (Fig. 6D). Furthermore, in early post-exponential phase, genes rank-ordered as the 12 most highly overexpressed in the GPL strain compared to the GP parent were also in the *paa* regulon (Data Set S4), so the functional importance of this upregulation in the absence of drug was pursued further. As confirmation of the overexpression of *paa*, RNA was isolated from parallel cultures of GP and GPL grown in the absence of drug and analyzed by quantitative reverse transcription PCR (q-rtPCR) (Materials and Methods). Consistent with the RNAseq results, transcript levels of the first gene of the operon, *paaA*, were significantly elevated in the GPL strain compared to the GP parent, with a 23-fold increase during exponential phase and a sixfold increase in early post-exponential phase (Fig. 7A and B). Another transcriptomic change of note in the GPL strain was the downregulation of the *atpE* gene encoding a component of the F_1_F_0_ proton translocating ATPase/synthase in the absence of drug (Fig. 6D). This parallels other work, which shows that depletion of the ATPase/synthase increases fitness of the GPL strain in the presence of CIP (35).

### Depletion of *paaA* alters expression of stress-related genes and lowers intracellular pH in the GPL pump hyper-expressor

The phenylacetate catabolic pathway breaks down the substrate phenylacetate to generate end products that feed into the TCA cycle (48). Previous studies indicate that induction of the *paa* pathway in *A. baumannii* may be a response to stress observed after antibiotic treatment (49). Therefore, upregulation of this operon may similarly support overproduction of the AdeL-regulated pump. It should be emphasized that *paa* mutants showed no significant fitness defect in our Tn-seq screen. Consistent with the Tn-seq findings, targeting *paaA* by CRISPRi caused no growth defect in the GPL strain as compared to a parallel non-targeting control strain. Depletion was efficient, as there was a 20-fold knockdown in exponential phase and almost 200-fold in early post-exponential phase compared to the negative control (Fig. 7C and D). This argues that in the absence of phenylacetate degradation, other compensatory pathways may allow fitness to be maintained.

The *paa* pathway has been shown to promote resistance to hydrogen peroxide (49, 50), so enhanced expression of the *paa* pathway in the AdeFGH pump hyper-expressor may facilitate a response to oxidative stress and/or other stressors. To test this idea, transcription of a set of oxidative stress genes was probed in the GPL strain by q-rtPCR analysis. Knockdown of *paaA* expression in early post-exponential phase yielded reduced transcript levels of *katG, katE*, and *sodC* (encoding two peroxidases and superoxide dismutase, respectively; Fig. 7D). This diminished expression is consistent with previous observations that *paa* null mutants are more sensitive to hydrogen peroxide stress through some previously uncharacterized mechanism (49, 50).

Intracellular pH was determined after *paaA* knockdown in the GPL mutant (Fig. 7E and F). Knockdown of *paaA,* the first gene of the large operon, resulted in a significant acidification during exponential growth compared to a non-targeting control (Fig. 7E) (A_600_ ~0.52–0.62). By early post-exponential phase (A_600_ ~1.22–1.39), the effect of the knockdown was no longer statistically significant compared to the non-targeting control (Fig. 7F). When the *paa* knockdown cultures were grown to exponential phase and exposed to sub-MIC levels of CIP for the last hour, the pHi was lower than the non-targeting control (Fig. 7G; significance determined via unpaired *t*-tests). These data indicate a role for this pathway in maintaining pH homeostasis during exponential growth of strains overexpressing the AdeL-regulated pump.

**Fig 8:**
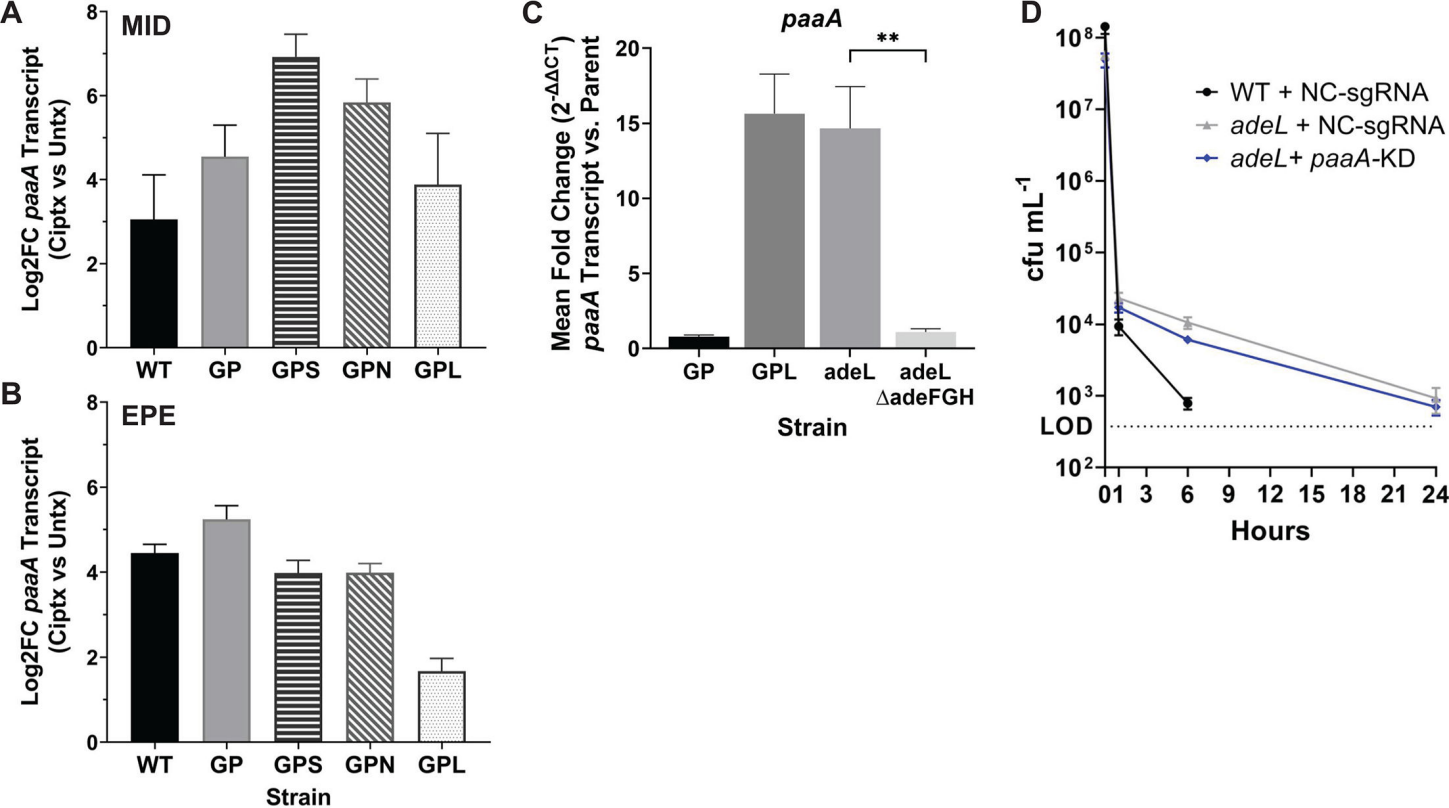
*paaA* is significantly upregulated during CIP exposure. (**A, B**) The *paaA* transcript (shown with L2FCSE) is upregulated after CIP exposure independently of strain background. Strains were grown to noted growth phases, with data derived from RNAseq experiments (Fig. 6; Materials and Methods). Significance determined by s values from triplicate cultures for each (s ≤ 1.11E − 03). (**C**) *paaA* upregulation is pump dependent. Strains were grown to mid-exponential phase and their RNA were used to quantify *paaA* transcript levels by q-rtPCR. Bars show mean transcript levels (2^-ΔΔCT^) ± SEM (*n* = 3) relative to parent strains (WT or GP), significance determined by ordinary one-way ANOVA (**, *P* = 0.0027). (**D**) Persistence of *adeL*∆I335A336 single mutant (in WT strain background) is unaffected by depletion of *paaA*. The indicated strains were grown to exponential phase with aTc inducer to deplete *paaA* transcript, then exposed to CIP at ~20× MIC for the noted times prior to plating on LB agar plates in the absence of drug to determine CFU/mL. The mean CFU/mL ± SEM are shown (*n* = 3). The dotted line indicates LOD, the limit of detection for the assay (375 CFU/mL). Mid, grown to mid-exponential phase; EPE, grown to early post-exponential phase; GP, *gyrA parC*.

Given that the *paa* operon has been documented to be associated with a drug stress response, we determined if the expression of the *paa* operon is altered in response to CIP in each of the strains. Based on the RNAseq data in exponential (Fig. 8A) and early post-exponential (Fig. 8B) phase, *paaA* transcript levels in all the strains were significantly upregulated in response to CIP exposure as compared to growth in the absence of drug (Fig. 8A and B). These results are consistent with previous results showing that upregulation of the *A. baumannii paa* catabolon occurs in response to drug stress (49). Notably, upregulation of *paa* transcript levels in the absence of drug is a special feature of the GPL strain when compared to the GP parent, and this is not observed in the GPS or GPN strains (Fig. 6D).

To determine if this upregulation is in response to overexpression of the RND efflux pump, rather than a consequence of a mutation in the regulatory element, the AdeL single mutant and AdeL mutant with the AdeFGH pump deleted were grown to mid-exponential phase with WT, GP, and GPL cultured in parallel. RNA was extracted and used for q-rtPCR to quantify *paaA* transcript levels. As anticipated, *paaA* transcript levels were elevated in the AdeL mutant. These levels dropped, however, in the absence of the AdeFGH RND efflux pump (Fig. 8C). This result supports the hypothesis that the *paa* operon is involved in a stress response to efflux pump hyperexpression.

Previous work has shown that pump hyperexpression and the oxidative stress response are contributors to the development of persistence in the presence of supra-MIC levels of antibiotics (51–54). In particular, the single AdeL mutant promotes persistence in the presence of about 20× MIC levels of CIP (54). We, therefore, determined if depleting *paaA* expression would interfere with persistence. Depleting *paaA* transcript did not alter the ability of a single *adeL* mutation in the WT background to persist in high levels of CIP when compared to the non-targeting control in AdeL (Fig. 8D). These results indicate that during exponential growth, upregulation of phenylacetate metabolism plays a role in maintaining cellular homeostasis in response to pump expression, mimicking the response of *A. baumannii* to antibiotic exposure.

## DISCUSSION

In this work, we set out to identify proteins that were required to maintain the fitness of *Acinetobacter baumannii* drug efflux pump-activated strains. The most surprising result was that in the AdeN and AdeS mutants, efflux pump overexpression was well-tolerated, with few mutations causing defects specific to these strain backgrounds (Fig. 3) and little compensatory transcriptional changes (Fig. 6). In contrast, the high-level FQR strain expressing the AdeL-regulated pump showed a range of mutations that lowered fitness relative to the WT control and a more pronounced shift in its transcriptome relative to a *gyrAparC* FQR mutant. An obvious difference between these mutants is that the *adeL*-activating mutations resulted in a higher level of expression of the AdeH outer membrane component than the corresponding AdeS- and AdeN-regulated outer membrane components (Fig. 6). The AdeS-regulated pump that was the most tolerant of insertion mutations has an outer membrane component that is not subject to AdeS-mediated upregulation (31, 32).

We previously observed that AdeL mutations confer persistence during high-level CIP exposure (54). In the mouse pneumonia model, *adeL*-activating mutants are outcompeted by strains that have increased fitness in the absence of antibiotic (54), consistent with *adeL-*activating mutations imparting a fitness cost. We suspect that the lowered growth rate is a consequence of envelope stress (Fig. 3) and could have clinical corollaries. The inability of *adeL* mutants to efficiently compete in immunocompetent animals (54) may mirror drug resistance evolution in hospitals, where immunocompromised patients may act as reservoirs for these variants that eventually act as evolutionary precursors to more fit and higher resistance variants. In this fashion, evolutionary precursors that cause cell envelope disruptions may be compensated by further mutations that allow spread in individuals with intact immune systems.

Fitness costs and resistance drivers are important for the evolution of MDR clinical isolates, and it is common for a pathogen to employ multiple drug efflux systems at a time, which has the potential for accumulated envelope stress (19). Of note, while it appears there is no advantage to overexpressing both AdeIJK and AdeFGH efflux systems simultaneously during CIP challenge (Fig. 3H; Fig. S17, GPNL mutant in Fig. S20), this was not investigated further in the presence of other drugs nor with AdeAB. This work does emphasize, however, that there is an interplay between fitness costs and fractional differences in drug resistance, and that strains having multiple antibiotic efflux systems may require compensatory mutations to reverse fitness costs. The work described here identified points of vulnerability in the high-level FQR strains that may be exploited to sensitize MDR strains to drug. It is evident that GPL heavily relies on determinants that stabilize the cell envelope and an effective alarmone response in the presence of drug. Furthermore, it may explain why *adeS-* and *adeN*-regulated pump overproducers appear to be particularly common in clinical isolates.

BfmS, identified in our Tn-seq screen, is the histidine kinase sensor that negatively regulates the BfmR response regulator in the BfmRS two-component regulatory system (TCS). BfmR controls a large transcriptional network including proteins linked to cell wall biogenesis, capsule production, and outer membrane protein assembly (42). It is intriguing that disruption of *bfmS* is detrimental to the fitness of the RND pump hyper-expressors, because disruption in a drug-sensitive isolate has been shown to increase drug resistance and the expression of genes such as *pbpG* that support RND pump expression (42). Presumably, proteins known to be preferentially downregulated or mis-localized when the TCS is activated by loss of BfmS may, in bulk, contribute to the fitness of the FQR strains. One such downregulated gene is *ompA*, encoding a major outer membrane protein of *A. baumannii* (55, 56), which apparently functions as a low-efficiency non-selective ion channel similar to *Escherichia coli* OmpA and *Pseudomonas* OprF (57, 58).

If there is a single feature linking together the Tn-seq mutant candidates, it is that they hit proteins associated with maintenance of outer membrane integrity. Various studies have demonstrated that defects causing instability to the outer membrane can be overcome by compensatory changes in envelope components, particularly associated with the outer membrane (59, 60). The functionality of the RND transporters is likely to be similarly supported by these components. We postulate that PG, LPS, and other OMPs are central players in allowing overproduction of the outer membrane component of the RND pump while simultaneously supporting outer membrane barrier integrity. As it provides a selective permeability barrier against antimicrobials, any destabilization of this barrier may simultaneously depress drug efflux pump function or lead to a compromised drug barrier, leading to drug sensitivity. Most of the insertions that caused fitness defects in pump-overexpressing strains were revealed only after the addition of CIP (Fig. 3). This means that either of these two mechanisms could come into play, with high pump concentrations combined with a partially dysfunctional outer membrane barrier collaborating to drive drug sensitivity.

There were gene disruptions that yielded significantly higher fitness in all FQR strain backgrounds when compared to the drug-susceptible WT strain, particularly after CIP exposure. The most striking knockouts were in *apaH,* encoding a dinucleoside polyphosphate hydrolase. ApaH degrades (p)ppGpp alarmones, which accumulate in response to stress as a consequence of RelA synthetase activity (61–63). Consistent with this result, we have recently shown that CRISPRi depletion of SpoT, which also degrades alarmones, increases fitness of GPL during CIP treatment (35). Further supporting the model that (p)ppGpp accumulation increases fitness during CIP treatment, transposon insertions in RelA, predicted to eliminate alarmone synthesis, resulted in a fitness reduction in the GPL background (Fig. S17A and C). Therefore, it is likely that accumulation of signals activating a stress response increases fitness specifically in the RND hyper-expressing strains, particularly in GPL.

The necessity of alarmone synthesis for optimum fitness was consistent with our observation that there were spikes in insertion mutation abundance at two prophage locations post-drug exposure in all strain backgrounds (Fig. S17 to 19C). These peaks were more prominent in the FQR strains, especially GPL, than in the WT strain and were responsible for many of the overrepresented insertions in these backgrounds (Fig. 3). Our previous work indicates that the abundance of these sequences is due to prophage induction after CIP exposure (17), and is associated with an amplified SOS stress response. Therefore, RND overproduction, particularly in the GPL strain, requires an effective stress response in the presence of CIP under conditions in which all strains are growing at equivalent rates.

Analysis of the transcriptome of the RND overproducers provides further evidence for the induction of cell stress as a consequence of AdeL-regulated pump overexpression (Fig. 6). Most notably, transcription of the phenylacetate operon (*paa*) was induced in response to pump overexpression in the absence of antibiotic treatment. This catabolon has been documented to protect against antimicrobial treatment (49) and supports virulence in *A. baumannii*, *Burkholderia cenocepacia,* and *Pseudomonas aeruginosa* (49, 64–66). Although the consequences of loss of *paaA* expression appear mild, there may be redundant pathways that come into play in the presence of drug pressure to support fitness of the AdeL mutant. The absence of *paaA* led to lowered cytoplasmic pH (Fig. 7E and G), so the production of substrates from the Paa pathway, which feed into the TCA cycle, could provide relief from proton influx produced by the overproduced pump. Alternatively, accumulation of phenylacetic acid could lead to lowered cytosolic pH upon knockdown of the *paa* operon. The importance of maintaining pH homeostasis in the presence of pump overproduction is further emphasized by the result that loss of the proton-pumping ubiquinol oxidase complex was predicted to cause lowered fitness of the GPL strain.

In summary, this work indicates that targets involved in supporting the integrity of the *A. baumannii* envelope are associated with maintaining fitness of strains that overexpress efflux pumps. The most highly expressed pump, regulated by AdeL, was associated with the largest number of genetic requirements for fitness, and was the most disruptive to the transcriptome. The genetic loci identified can be viewed as drug targets for synergy with fluoroquinolones as well (Fig. S21), defining pathways that may buffer the stress that accompanies increased pump activity. Still unclear is how the phenylacetate degradation pathway is induced in response to the stress identified here, and what physiological role, if any, it plays in supporting the fitness of the strains analyzed in this study. Future work will attempt to expand these studies into analyzing the role of essential proteins in supporting pump overproduction in hopes of identifying potential targets for multidrug therapy targeting drug-resistant mutants.

## MATERIALS AND METHODS

### Bacterial strains and growth conditions

Bacterial strains used in this work are listed in Table S1. All *Acinetobacter baumannii* strains are derived from ATCC 17978UN. Cultures were grown in LB (10 g/L tryptone, 5 g/L yeast extract, 10 g/L NaCl) or on LB agar plates (LB supplemented with 15 g/L agarose) and incubated at 37°C with aeration, as well as shaking, or on a roller drum if grown in broth. Unless grown the same day, cultures were typically diluted, incubated to recover after overnight growth, then diluted to A_600_ = 0.1–0.001 into either 5 mL–10 mL broth or into 96-well plates (Costar), and growth was monitored by measuring A_600_ every hour (broth culture) or every 15 min (Epoch 2 or SynergyMx, [Biotek] plate readers.) Growth is then plotted as log-transformed absorbance values, with the mean ± SEM shown. Superoptimal broth with catabolite repression (SOC) was used for recovery following all electroporations of *E. coli* and *A. baumannii* during cloning or library preparations. Electroporations of *A. baumannii* were performed using 0.1 cm gap cuvettes (0.2 cm for *E. coli*) and the Gene Pulser (Bio-Rad) at the settings 200 Ω, 25 µF, and 1.8 kV (at 2.2kVd). As needed, LB was supplemented with 10% sucrose or antibiotics at the following concentrations: ampicillin: 100 µg/mL; carbenicillin: 50 µg/mL–1,000 µg/mL; gentamicin (Gent): 100 µg/mL; chloramphenicol: 100 µg/mL; kanamycin (Kan): 20 µg/mL; or CIP: varying concentrations (Sigma).

### Molecular cloning and mutant construction

Primers (IDT) and plasmids described in this study as well are listed in Table S1. The AdeS (R152S) mutant was isolated by drug selection of a precursor strain by plating on LB with 10 µg/mL gentamicin. Single colonies were purified on LB agar plates, and then *adeR* and *adeS* were amplified by colony PCR and sequenced (Genewiz) to identify mutations. The selected *adeS* mutation (R152S) was introduced into *A. baumannii gyrA(S81L) parC(S84L)* [also called GP (GVA37)] to create *gyrA(S81L) parC(S84L) adeS(R152S*) in a fashion similar to that described previously [also called GPS (EHA105)] (67). Briefly, the mutant allele was amplified with upstream and downstream flanking sequences to generate a product of about 1,500 bp in length, and introduced first into pUC18 by restriction enzyme digestion and T4 DNA ligation. Once propagated in DH5α and sequence confirmed (Genewiz), the mutant allele was introduced into the pJB4648 plasmid by restriction enzyme digestion and T4 DNA ligation and propagated in DH5α λpir. The mutant allele in pJB4648 was then introduced into *A. baumannii* by electroporation and homologous recombination with two selection steps, the first selecting for transformants on LB + 10 µg/mL gentamicin on bacteriological plates and the second counterselection for Gent^S^ on LB + 10% sucrose plates at either room temperature or at 30°C. The internal, in-frame gene deletion mutants were previously created or constructed during this study using the same methodology after three-way restriction enzyme digestions and ligations (41, 42, 67). The GPL (EHA23) strain [*gyrA(S81L) parC(S84L) adeL(∆I335A336*)] was constructed using the gentamicin selection/sucrose counterselection. The *adeL(∆I335A336*) allele was PCR amplified from gDNA isolated from P9 Cy3.2 (54) with flanking regions, cloned into pUC18 then moved into pJB4648, propagated in XL1 Blues and DH5α λpir *E. coli*, sequence confirmed, then introduced into the GP background by electroporation and homologous recombination. GP was created previously (17) and *gyrA(S81L) parC(S84L) adeN*::ISAba1 (GVA47), also known as GPN, was generated by plating GP on LB + 32 µg/mL CIP.

### WGS

Strains used in this study were sequence verified by WGS as previously described (54). Illumina Nextera reagents were used for library preparation and 100 bp single-end reads were obtained using the Tufts University Core Facility’s HiSeq 2500. BRESEQ 0.35.5 was used to align reads to the *Acinetobacter baumannii* 17978UN reference genome (GenBank accession: CP079212.1), and mutations, those of which are absent in the parent strains, were identified. All sequences are deposited in the SRA database under BioProject number PRJNA1068501.

### LOS analysis

LOS profiles were examined using tricine gel electrophoresis of cell lysates as previously described (41), using lysates from cells grown to mid-logarithmic phase. After fractionation on Novex 16% tricine gels (Invitrogen), the gels were stained with Pro-Q Emerald 300 (Invitrogen) for LOS staining, and Coomassie brilliant blue for total protein staining for sample quantification normalization (41).

### pHi assay

Buffers of varying pH were made using 50 mL combinations of 0.2M Na_2_HPO_4_ and 0.2M NaH_2_PO_4_ (68). Also, 5 mM BCECF/AM (Thermo Fisher) and 0.5 mM CCCP (Sigma) were prepared in dimethyl sulfoxide (DMSO) and stored at −20°C until use. Methods were adapted with modifications from previous protocols (69). Overnight cultures prepared from individual colonies were diluted for recovery to allow growth, then diluted again to A_600_ ~0.003 in 8 mL LB (with or without drug as indicated). In the case of dCas9 strains, single colonies were inoculated into 2 mL LB in capped tubes and incubated to late exponential phase before diluting into LB + 100 ng/mL aTc. The cultures that were exposed to sub-MIC levels of CIP were treated with 45 µg/mL of CIP an hour prior to harvest. Cultures were grown to exponential phase (A_600_ = 0.4–0.7) or early post-exponential phase (A_600_ = 1.2–1.4), after which samples (about 5 mL) were pelleted in 15 mL conical tubes at 7,500 g for 5 min, resuspended in sterile phosphate-buffered saline (PBS, Thermo Scientific), loaded with 20 µM BCECF/AM in PBS followed by incubation at 30°C for 30 min in a 96-well v-bottom plate protected from light.

Cells in the 96-well plate were diluted 1:4, pelleted at 2,000 rpm for 5 min, resuspended in LB, and incubated at 37°C for 5 min, before pelleting once more and re-suspending in 200 µL–400 µL previously prepared mono- and di-basic sodium phosphate buffer at neutral pH (experimental samples) or at a range of pH values (*in situ* standard curve samples). Samples were diluted 1:1 in the neutral or pH-adjusted buffers to a final volume of 200 µL in a 96-well black plate with a clear, flat bottom (Costar). About 10 µM CCCP stock was added to the *in situ* standard curve wells. All samples were mixed via pipetting before fluorescence was read at 490ex/535em and 440ex/535em after 5 min (Biotek SynergyMx with Gen5 software). Background fluorescence from buffer alone was subtracted from each sample before calculating the ratiometric fluorescence of the calibration curve samples and experimental samples. The experimental sample intracellular pH was interpolated from the *in situ* calibration curve using standard curve fitting.

### Preparation of transposon mutant banks

Transposon banks with *Himar-1* transposon were created as described (17, 41). Fifty microliters of electrocompetent *A. baumannii* cells was electroporated with 300 ng pDL1100 (*Himar-1-*Kan^R^). Cells were recovered in 1 mL SOC broth, placed in 14 mL snap cap tubes (Corning), and aerated on a drum roller for 15 min at 37°C. The cells were pelleted and resuspended in 400 µL SOC, then spread onto two 150 mm culture dishes containing mixed cellulose ester filters (0.45 µm pore size, 137 mm diameter; Advantec) that had been placed on top of the SOC agar. Plates were incubated for 1 hour at 37°C before filters were transferred to 150 mm culture dishes containing LB + 20 µg/mL kanamycin and incubated overnight (~16 hours) at 37°C.

Mini-*Tn10* banks were also made in the GPN strain background. GPN electrocompetent cells were transformed with 75 ng pDL1073 (mTn*10-neo*), with subsequent steps following the same procedure as used for *Himar-1*. CFU counts were determined from plate images using an Image J counting routine. Mutants were pooled from filters by gentle shaking in 50 mL–75 mL sterile PBS. Glycerol was added for a final concentration of around 10% and aliquots stored at −80°C until use. About 21 independent subpools were created in each strain background (producing around 100,000–150,000 cumulative unique mutants).

### CIP challenge of transposon banks

Independent subpools comprising approximately 7,500–16,000 unique transposon mutants were challenged with antibiotic as described (17, 41). Mutant banks were thawed on ice, diluted in 25 mL LB to an A_600_ = 0.1, grown to A_600_ = 0.2 at 37°C, then diluted to A_600_ = 0.003 in 10 mL LB with or without CIP of varied concentrations added in parallel cultures. The remaining culture (T_1_) was harvested to determine what mutants were present prior to outgrowth and drug challenge. Cultures were incubated for about eight generations (around 170 min if untreated, 225 min if CIP treated), then harvested (T_2_). Drug-treated cultures that exhibited 20%–30% growth inhibition were harvested. Cultures were pelleted and stored at −20°C until all samples were collected. The concentration ranges at which comparable growth inhibition was met for each strain background as well as the average population expansions between T_1_ and T_2_ with each condition (according to A_600_) are noted in Data Set S2.

### Preparation of transposon libraries for sequencing

As described (17, 41), the gDNA was extracted using the DNeasy Blood and Tissue Kit (Qiagen). Samples were treated with proteinase K during resuspension and incubated at 56°C with shaking for 1 hour before treatment with RNase cocktail (Fisher Scientific) for 8 min at 62°C. The gDNA was quantified with SYBR green then diluted to 7 ng/µL in UltraPure water (Fisher Scientific). About 28 ng of gDNA of each sample was tagmented using TD buffer and TDE1 enzyme (Illumina) and incubated in a thermocycler at 55°C for 5 min prior to melting at 95°C for 0.5 min. Transposon insertion sites were then amplified using Q5 hot start polymerase (NEB) and 0.6 µM Nextera 2 A-R and olj638 (for *Himar-1* libraries), or olj928 (for Tn*10* libraries). The 50 µL reactions were incubated at 98°C for 1 min, cycled 30 times through 98°C for 10 s, 65°C for 20 s, and 72°C for 1 min, then finally at 72°C for 2 min. In addition, 0.5 µL of the first PCR products was used for a second PCR reaction used to introduce indexes with leftward *Himar-1-* or Tn10-specific indexed primers and rightward Nextera indexed primers. The reaction and settings were the same except cycling was shortened to 12 cycles. Amplified products were quantified via 2% agarose tris-acetate-EDTA gels using SYBR Safe (Invitrogen) for visualization. The gels were loaded with a known amount of 100 bp DNA ladder (NEB) alongside samples of each reaction to quantify their concentrations based on band intensity around 250 bp–600 bp using a gel imaging system. Equal measures of all samples were thereafter pooled and purified via PCR column clean-up (Qiagen). About 17.5 pmol of each pooled sample was reconditioned in an additional 50 µL reaction with primers P1 and P2. Thermocycler settings were set with a heated lid and incubations at 95°C for 1 min, with slow ramp of 0.1°C/s to 64°C, then held for 20 s, and finally 72°C for 10 min. The products were purified once more with the PCR column clean-up kit (Qiagen). Primer olj115 (Tn*10*) or mar512 (*Himar-1*) was used for sequencing at the Tufts University Core Facility of Genomics with the Illumina HiSeq 2500 (SE50, post PippinPrep (Sage Science) size selection for 250 bp–600 bp).

### Sequence analysis and assessment of mutant fitness

Sequenced reads were processed and analyzed as done previously (17, 40, 41, 70). After demultiplexing, the Nextera adapter sequence was clipped from reads using the fastx_clipper, quality filtered, and reads shorter than 20nt were discarded. Reads were mapped to the *A. baumannii* ATCC 17978 reference genome (https://genomes.atcc.org/genomes/e1d18ea4273549a0) using Bowtie 1.2.2, formatted so only unique and best-mapped reads were aligned, allowing up to one maximum mismatch. Fitness scores were then calculated for each transposon mutant using MAGenTA (70), with scores averaged and aggregated at the gene level across replicates. Insertions within 10% of 3´ end of genes were excluded, and fitness scores normalized to a set of neutral/pseudo genes. Of note, fitness scores of GPN insertion mutants were determined using data from both *Himar-1* and mini-*Tn10* banks. All other results were from *Himar-1* mutagenesis. Fitness scores of gene disruptions in all strain backgrounds were normalized to a LOWESS curve to avoid the position bias exhibited post CIP exposure. The difference in fitness scoring for each gene disruption was assessed between each FQR strain and the WT drug-susceptible strain. Significant fitness differences yielded from the same gene disruption between the different strain backgrounds were identified via multiple *t*-test analysis using the original false discovery rate (FDR) method of Benjamini and Hochberg with significance falling within the FDR of 5% (q < 0.05).

### RNAseq experiment to uncover transcriptional differences between FQR and parent strains

Cultures were grown overnight, diluted to an A_600_ of 0.1 in 10 mL LB, grown for about 1.5 hours at 37°C on a drum wheel to approximately mid-log growth, then diluted once more to A_600_ of 0.003 in fresh LB with or without CIP. Some of these were grown to approximately mid-log phase by incubation for 150 min–165 min (untreated) or for 195 min–225 min (CIP treated) as similarly done before (17) and reaching A_600_ ~0.445–0.68 and A_600_ ~0.39–0.66, respectively. Others were grown for 195 min–210 min (untreated) reaching A_600_ ~0.85–1.195 or for 255–270 min (CIP treated) to A_600_ ~0.82–1.125. Of the cultures treated with CIP, those that exhibited 20%–30% growth inhibition (compared to the untreated culture grown in parallel) were harvested (details provided in Data Sets S3 and S4). About 1–1.5 A_600_ units of all cultures were harvested and frozen at −80°C until all samples were collected.

### Preparing RNA for sequencing

RNA was isolated from the samples using the RNeasy Mini kit (Qiagen) and prepared for sequencing as described (17, 71, 72) following published protocols (73). Approximately 600 ng of RNA was fragmented at 94°C with 10× FastAP buffer for 2.5 min then treated with TURBO DNase, FastAP (Thermo Scientific) (and murine RNase inhibitor; NEB) for 30 min at 37°C. Samples were then purified using Agencourt RNAClean XP (Beckman Coulter) and eluted with 12.5 µL of DEPC UltraPure water (Fisher Scientific). Five microliters of sample was incubated with 1 µL of 100 µM barcoded RNA adapters at 72°C for 2 min before being mixed with DMSO (American Bioanalytical), 10× T4 RNA ligase buffer, 100 mM ATP, RNase inhibitor murine, T4 RNA ligase, and 50% PEG8000 (NEB) to allow for 3´ linker ligation at room temperature for 1.5 hours. The 60 samples were pooled into five groups of 12 samples each during purification using RNA Clean & Concentrator (Zymo Research), and all were eluted with 16 µL DEPC UltraPure water.

Ribosomal rRNA was depleted using RNase-H as described (74). Probes complementary to *A. baumannii* ATCC 17978UN 23S, 16S, and 5S rRNA sequences (CP079931.1) were created using 0.design_probe.py and off-target binding predicted using 2.predict_probe_offtarget.py (https://github.com/hym0405/RNaseH_depletion). The 89 devised oligonucleotides (IDT) were resuspended into 100 µM in UltraPure water, and a master-mix was created with 5 µL of each probe. For each depletion, the RNA was mixed with 1 µL of the probe mixture, 0.6 µL 1M Tris-HCl pH 7.5, 1.5 µL 5M NaCl, and DEPC UltraPure water for a final 15 µL reaction. They were incubated in a thermocycler with a heated lid at 95°C for 2 min, temperature reduced to 45°C in 0.1°C/s increments and incubated a further 5 min at 45°C. Meanwhile, RNase-H master mix was prepared for each depletion (3 µL Hybridase Thermostable RNase H; Epicentre), 0.5 µL 1M Tris-HCl pH = 7.5, 0.2 µL 5M NaCl, 0.4 µL 1M MgCl_2_, and 0.9 µL DEPC UltraPure water) and preheated to 45°C. Post probe and RNA hybridization, 5 µL prewarmed RNase-H mixture was added to each probe-RNA mixture and incubated at 45°C for 30 min. These were then purified twice with 2× SPRI RNAClean XP (Agencourt) and eluted with 13 µL DEPC UltraPure water.

The RNA preparation was then used as a template for cDNA synthesis. Twelve microliters of sample was incubated with 2 µL of 25 µM AR2 primer at 72°C for 2 min using 10× AffinityScript RT buffer and enzyme (Agilent Technologies), 0.1M dithiothreitol (DTT), 100 mM deoxynucleotide triphosphates (dNTPs), and 40 U/µL murine RNase inhibitor. After 55 min at 55°C, RNA was then denatured in a 10% reaction volume (adding 2 µL to the 20 µL cDNA reaction product) of 1N freshly prepared NaOH at 70°C for 12 min before neutralization with 4 µL of freshly prepared 0.5M acetic acid. The products were then subjected to Agencourt RNAClean XP, eluted with 5 µL DEPC UltraPure water, incubated with 2 µL of 40 µM 3Tr3 adaptor at 75°C for 3 min with the beads still present, and mixed with 10× T4 ligase buffer, DMSO, ATP, PEG8000, and T4 RNA ligase for an overnight 20 µL ligation reaction at room temperature. The products were purified with SPRI RNAClean XP (Agencourt) beads twice and finally eluted with 25 µL H_2_0. DNA was amplified from cDNA using 10 µL of 5× Q5 reaction buffer and 2.5 µL Q5 enzyme (NEB), 0.5 µL 100 mM dNTPs, 10 µL cDNA, 2.5 µL primers RTS_Enr_P5 and RTS_Enr_P7_BCXX, and water for 50 µL reaction. The reactions were incubated at 98°C for 3 min, cycled at 98°C for 30 s, 67°C for 25 s, and 72°C for 15 s for 14 cycles, and completed at 72°C for 2 min. PCR products were purified once with AxyPrep Magnetic PCR beads (Axygen; Corning). DNA was then quantified by Qubit with the dsDNA HS Assay kit (RNA HS Assay kit was used previously for the quantification of the RNA samples; Thermo Scientific) and about 100 nM of each of the five subpools was pooled together into one sample for sequencing.

Throughout sample preparation, 1 µL SUPERase-IN RNase inhibitor (Thermo Scientific) was added to samples if they were stored at −80°C in between steps. RNAse Zap was used continuously on surfaces until cDNA was synthesized. Samples were routinely analyzed by submitting samples for QC fragment analysis at the TUCF Genomics Core. The pooled samples were sequenced at this facility, using a NextSeq 550 Single-End High output, with 75 cycles following nanoQC.

### RNAseq analysis

Sequences that had been de-multiplexed for the i7 barcodes were then de-multiplexed for the i5 barcodes using fastx_barcode_splitter.pl (https://github.com/agordon/fastx_toolkit/). The 5´ anchored adaptor sequences were then trimmed *in silico* using Cutadapt (75). Truseq adaptor sequences were also trimmed *in silico*, disallowing any error, and dropping resulting sequences shorter than 20nt. Sequence data were thereafter analyzed following and modifying published scripts (https://github.com/huoww07/Bioinformatics-for-RNA-Seq). The Acinetobacter_baumannii_ATCC_17978.fasta and Acinetobacter_baumannii_ATCC_17978.gbk files were downloaded from the ATCC genome portal (https://genomes.atcc.org/genomes/e1d18ea4273549a0?tab=overview-tab) to create the STAR genome index (via genomeGenerate with STAR). Annotation GTF files were constructed using Geneious, then cufflinks gffread for conversion to a strict gtf file. Alignments were then performed using STAR. Subread/1.6.3 was used to get a summary of the sequence data features, then featurecount_stat.R as well as mapping_percentage.R were adapted to analyze and visualize features including mapping efficiency and overview of mapped vs unmapped reads. Using the Tufts Cluster on-demand R studio app, DESeq2, ggplot2, dplyr, tidyverse, ggrepel, DEGreport, and pheatmap tools were employed to analyze the quality of the data set as well as visualize and deconvolve the contrasting transcriptomic data between the high-level FQR pump-overproducing strains and the GP parental strain.

### qPCR analysis for transcript abundance

Cultures derived from individual colonies were grown in LB to mid- or early post-exponential phase. About 1 A_600_ unit of culture was harvested, and RNA was extracted using RNeasy (Qiagen). cDNA was synthesized after ezDNase treatment using the SuperScript VILO cDNA kit (Thermo Fisher), then amplified using PowerUp SYBR Green Master Mix (Applied Biosystems) and StepOnePlus Real-Time PCR system (Applied Biosystems) per manufacturer’s instructions. The efficiency of target amplification was tested using a standard curve of cDNA at varying dilutions and consequently verified to be greater than 95% for each primer pair used. Three biological replicates were evaluated for each strain and three technical replicates examined per biological sample. To confirm a lack of gDNA contamination, control samples tested in parallel which lacked reverse-transcriptase during cDNA synthesis were included. Transcript levels for each indicated target were compared between each mutant strain and the parental strain (via the comparative 2^-ΔΔCT^ method) after normalization to that of 16S, the endogenous control. The data are represented in bar plots showing the mean fold difference in transcript levels (2^-ΔΔCT^) ± SEM.

### MIC determination

MICs were determined by broth microdilutions. Overnight cultures were diluted to A_600_ = 0.1 in 3 mL LB, incubated to early exponential phase (A_600_ = 0.2–0.3), then diluted to a final A_600_ = 0.003 in 200 µl LB in the presence of different dilutions of CIP in a 96-well plate (Costar). Growth was monitored by measuring A_600_ every 15 min (Epoch 2 or SynergyMx, [Biotek] plate readers). At least three replicates were tested for all strains. CIP MIC was determined as the lowest concentration that prevented growth up to 12 hours, where geometric mean A_600_ units did not exceed 0.035, and are not significantly different from the LB-only negative controls. CIP MICs were 256 µg/mL (GPS and GPN) and 384 µg/mL (GPL).

### Persistence assay

The *A. baumannii* strains EHA417 [*adeL*∆I335A336 *P_Tet_::dCas9* (pWH1266-*paaA*-sgRNA)], EHA418 [*adeL*∆I335A336 *P_Tet_::dCas9* (pWH1266-NC-sgRNA)], and EHA254 [WT *P_Tet_::dCas9* (pWH1266-NC-sgRNA)] were incubated in 1.5 mL LB in triplicate for 2–3 hours. They were then diluted 1/1,000 in 8 mL LB + 100 ng/mL aTc to induce dCas9 expression and grown to mid-exponential phase. Each culture was then normalized to A_600_ ~0.5 with fresh LB containing inducer aTc, then treated with 10 µg/mL CIP. Samples of culture (500 µL) were removed at timepoints = 0, 1, 6, and 24 hours, washed with PBS, then resuspended in PBS, and serial dilutions were spotted on LB agar plates for overnight incubation at 37°C and CFU enumeration the next day.

### Microscopy

Culture samples were placed on 1% agarose in PBS pads and imaged using differential interference contrast (DIC) at 100× magnification on the Zeiss Axiovert Observer.Z1.

## Data Availability

All analyzed sequences are in the SRA database under BioProject numbers PRJNA1067619 (TnSeq), PRJNA1067535 (RNAseq), and PRJNA1068501 (WGS).

## SUPPLEMENTAL MATERIAL

Data Set S1 mbio.03221-24-s0001.xlsx TRANSIT resampling data with comparisons made between FQR and WT strains.

Data Set S2 mbio.03221-24-s0002.xlsx TnSeq data with fitness comparisons made between FQR and WT strains.

Data Set S3 mbio.03221-24-s0003.xlsx RNAseq data with comparisons made for transcriptomes between the FQR and GP parent strains during CIP exposure.

Data Set S4 mbio.03221-24-s0004.xlsx RNAseq data with comparisons made for transcriptomes between the FQR and GP parent strains in the absence of drug treatment.

Supplemental Figures mbio.03221-24-s0005.pdf Figures S1 to S21.

Legends mbio.03221-24-s0006.docx Supplemental material legends.

Table S1 mbio.03221-24-s0007.xlsx Bacterial strains, plasmids, and primers used in this study.

ASM does not own the copyrights to Supplemental Material that may be linked to, or accessed through, an article. The authors have granted ASM a non-exclusive, world-wide license to publish the Supplemental Material files. Please contact the corresponding author directly for reuse.
