# Supplementary material for: Identification of determinants that allow maintenance of high-level fluoroquinolone resistance in Acinetobacter baumannii
Source: mBio. 2024 Nov 26;16(1):e03221-24. doi: 10.1128/mbio.03221-24 (PMC11708032; doi:10.1128/mbio.03221-24)
Supplement: Supplemental Figures — Figures S1 to S21. [file mbio.03221-24-s0005.pdf]

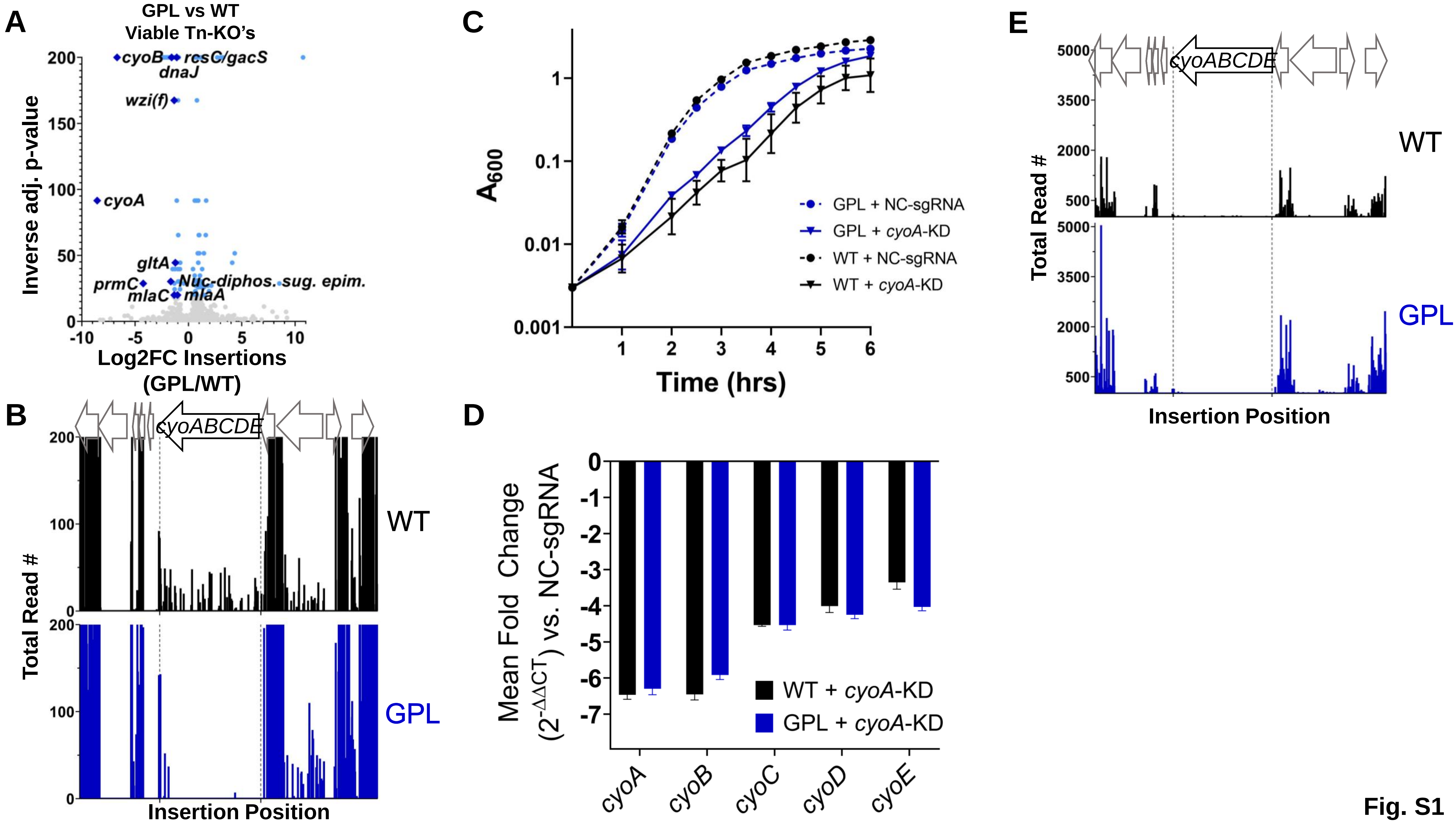
A
GPL vs WT
Viable Tn-KO's
Inverse adj. p-value
cyoB
resC/gacS
dnaJ
wzi(f)
cyoA
gltA
prmC
Nuc-diphos. sug. epim.
mlaC
mlaA
Log2FC Insertions
(GPL/WT)
B
cyoABCDE
WT
GPL
Total Read #
Insertion Position
C
A600
Time (hrs)
GPL + NC-sgRNA
GPL + cyoA-KD
WT + NC-sgRNA
WT + cyoA-KD
D
Mean Fold Change
(2-ΔΔCT) vs. NC-sgRNA
WT + cyoA-KD
GPL + cyoA-KD
cyoA
cyoB
cyoC
cyoD
cyoE
E
cyoABCDE
WT
GPL
Total Read #
Insertion Position

Fig. S1

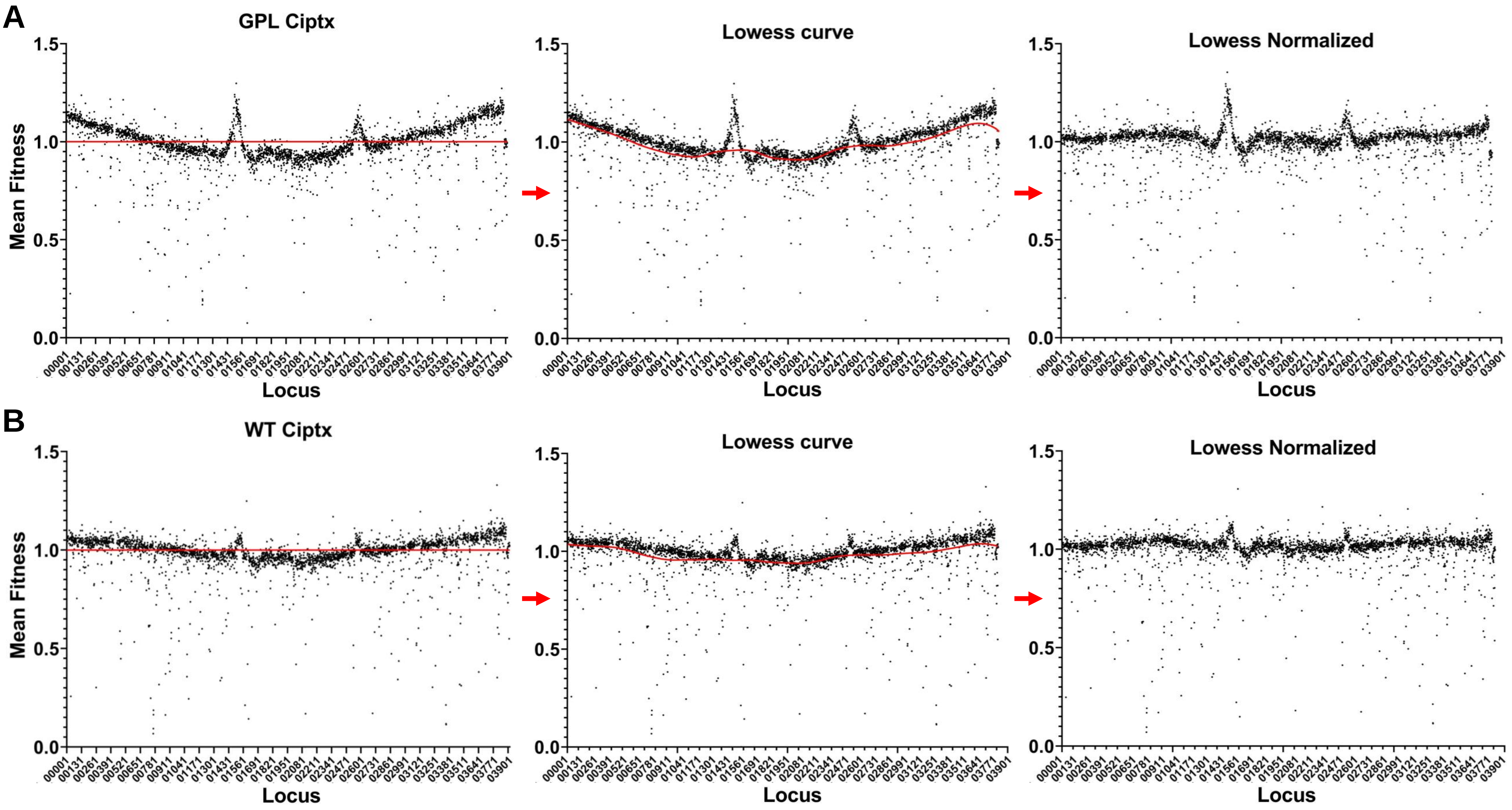
A
GPL Ciptx
Lowess curve
Lowess Normalized
Mean Fitness
Locus
1.5
1.0
0.5
0.0
00001
00131
00261
00391
00521
00651
00781
00911
01041
01171
01301
01431
01561
01691
01821
01951
02081
02211
02341
02471
02601
02731
02861
02991
03121
03251
03381
03511
03641
03771
03901
B
WT Ciptx
Lowess curve
Lowess Normalized

Fig. S2

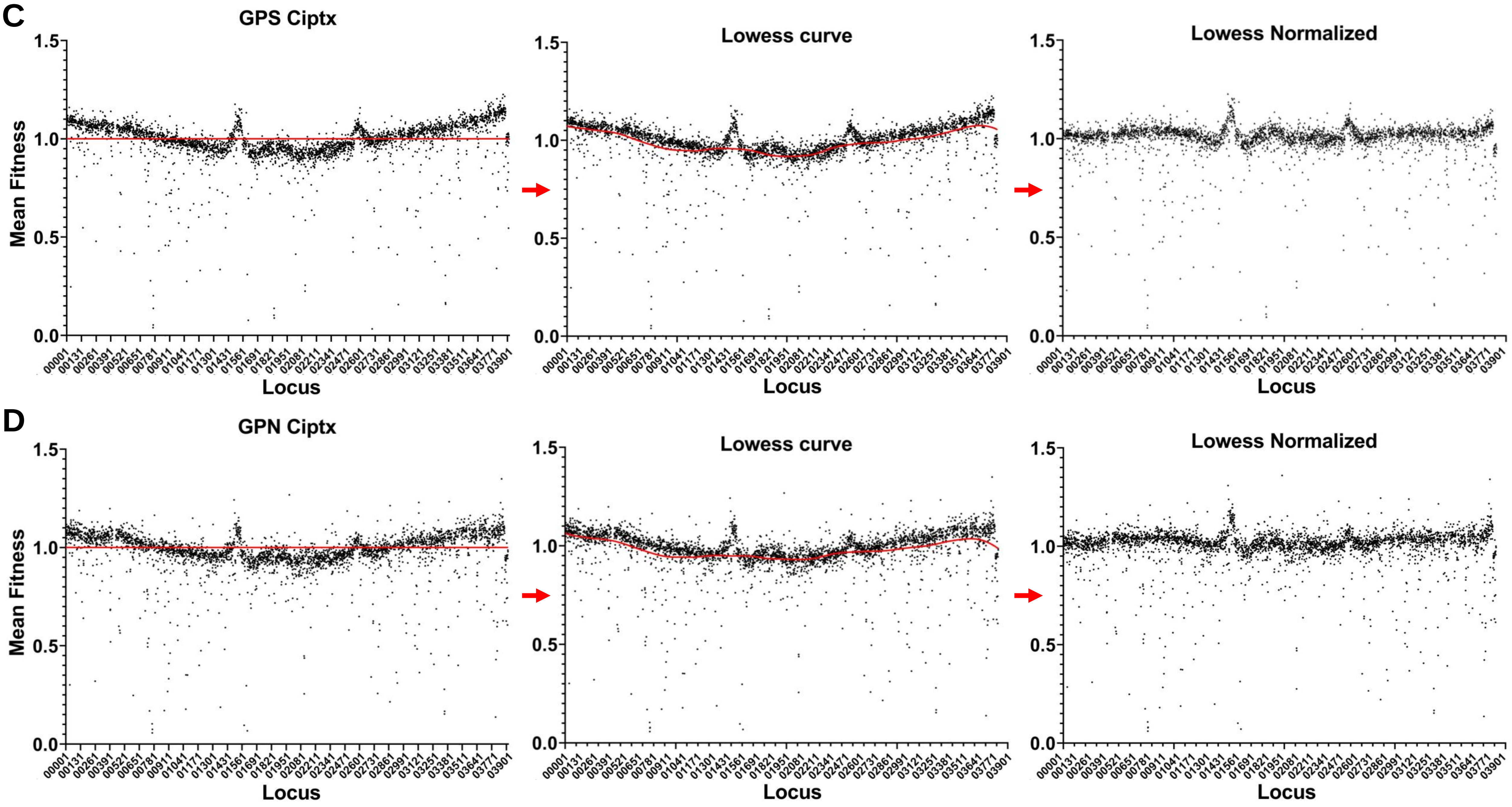
C
GPS Ciptx
Lowess curve
Lowess Normalized
D
GPN Ciptx
Lowess curve
Lowess Normalized
Mean Fitness
Locus
0.0
0.5
1.0
1.5
00001
00131
00261
00391
00521
00651
00781
00911
01041
01171
01301
01431
01561
01691
01821
01951
02081
02211
02341
02471
02601
02731
02861
02991
03121
03251
03381
03511
03641
03771
03901

Fig. S2

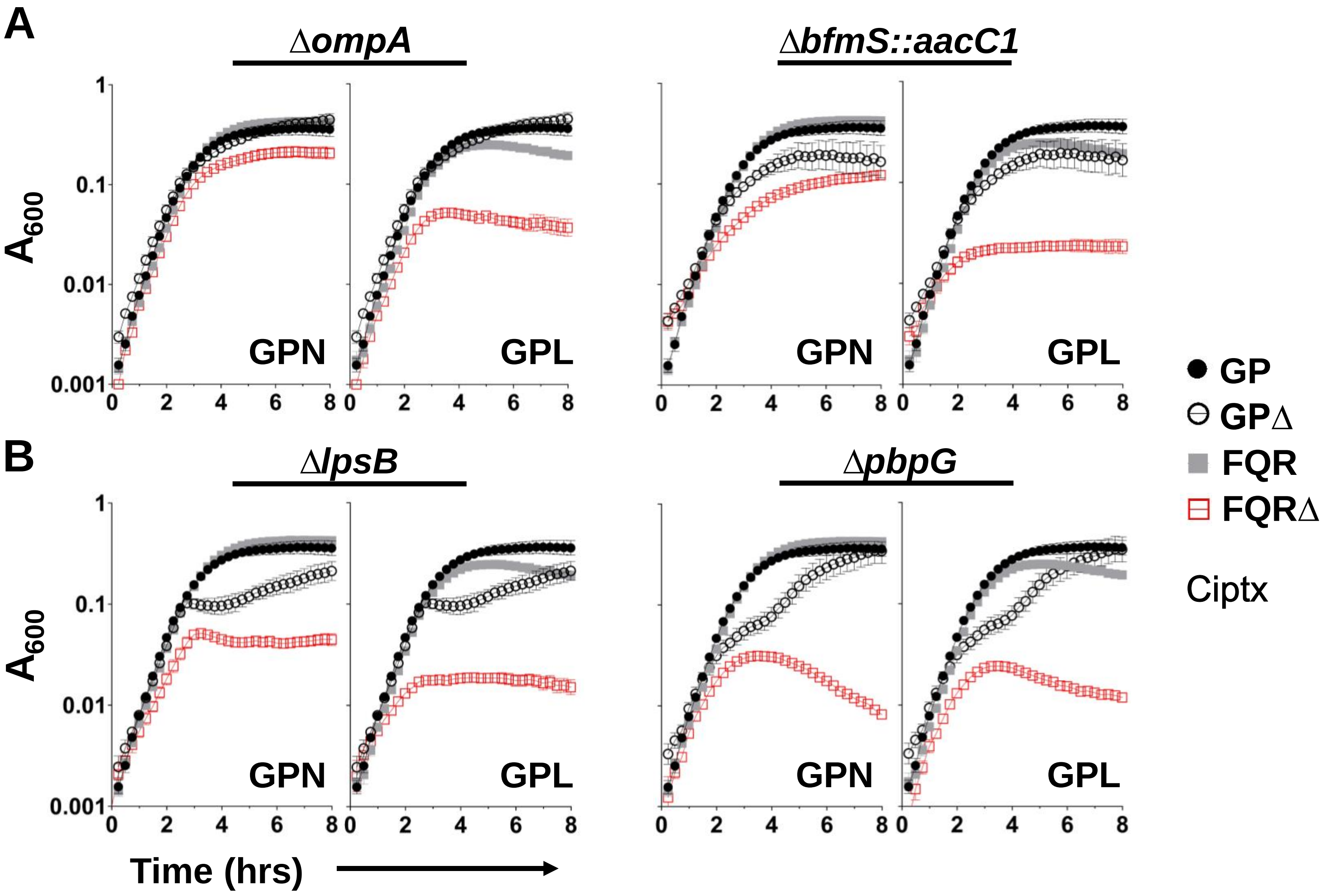

A
ΔompA
ΔbfmS::aacC1
B
ΔlpsB
ΔpbpG
A600
1
0.1
0.01
0.001
0
2
4
6
8
GPN
GPL
Time (hrs)
GP
GPΔ
FQR
FQRΔ
Ciptx

Fig. S3

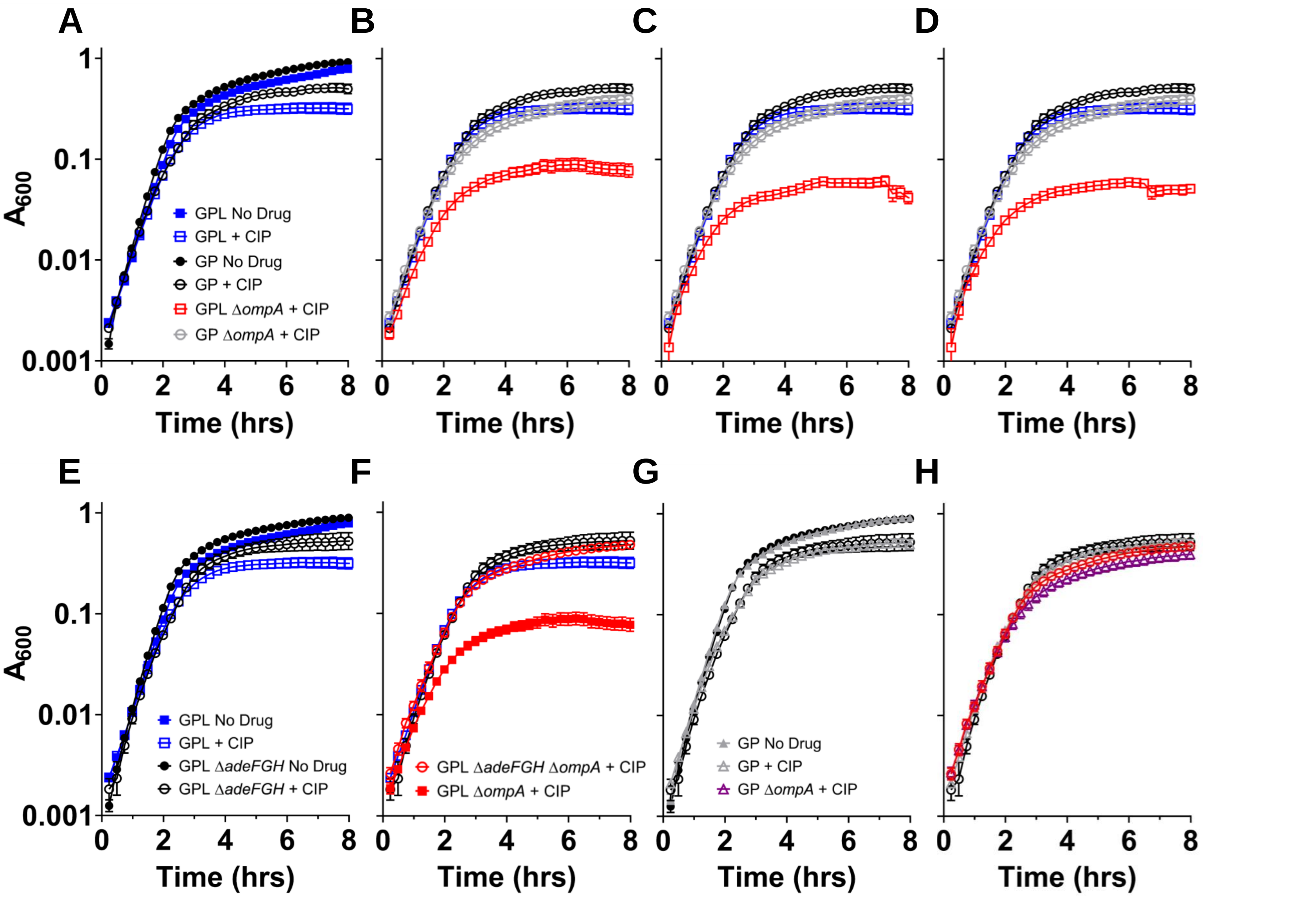
A
B
C
D
E
F
G
H
A600
Time (hrs)
GPL No Drug
GPL + CIP
GP No Drug
GP + CIP
GPL ΔompA + CIP
GP ΔompA + CIP
GPL ΔadeFGH No Drug
GPL ΔadeFGH + CIP
GPL ΔadeFGH ΔompA + CIP
GPL ΔompA + CIP
GP No Drug
GP + CIP
GP ΔompA + CIP

Fig. S4

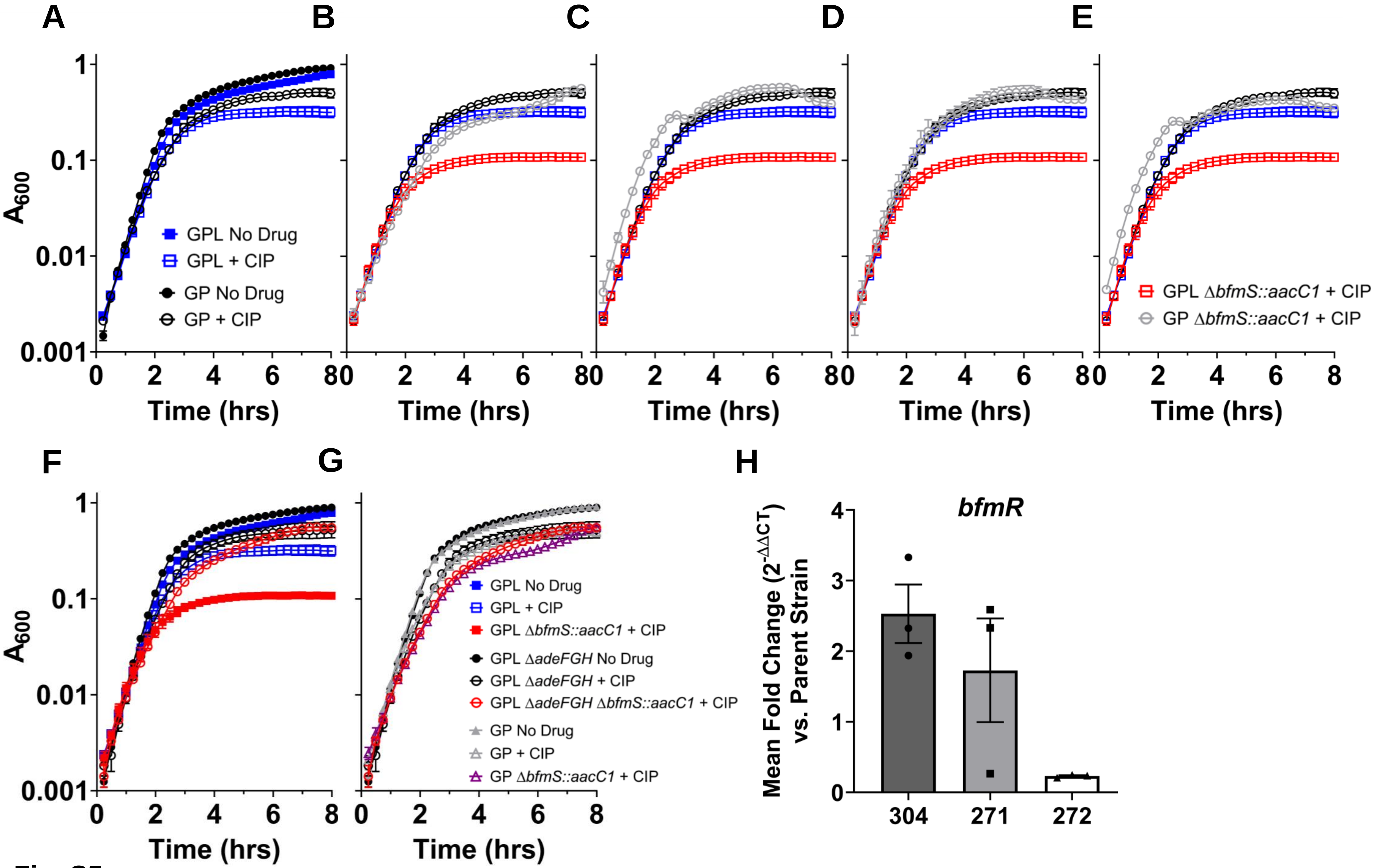
A
B
C
D
E
A600
GPL No Drug
GPL + CIP
GP No Drug
GP + CIP
GPL ΔbfmS::aacC1 + CIP
GP ΔbfmS::aacC1 + CIP
Time (hrs)
F
G
H
GPL No Drug
GPL + CIP
GPL ΔbfmS::aacC1 + CIP
GPL ΔadeFGH No Drug
GPL ΔadeFGH + CIP
GPL ΔadeFGH ΔbfmS::aacC1 + CIP
GP No Drug
GP + CIP
GP ΔbfmS::aacC1 + CIP
bfmR
Mean Fold Change (2^-ΔΔCT) vs. Parent Strain
304
271
272

Fig. S5

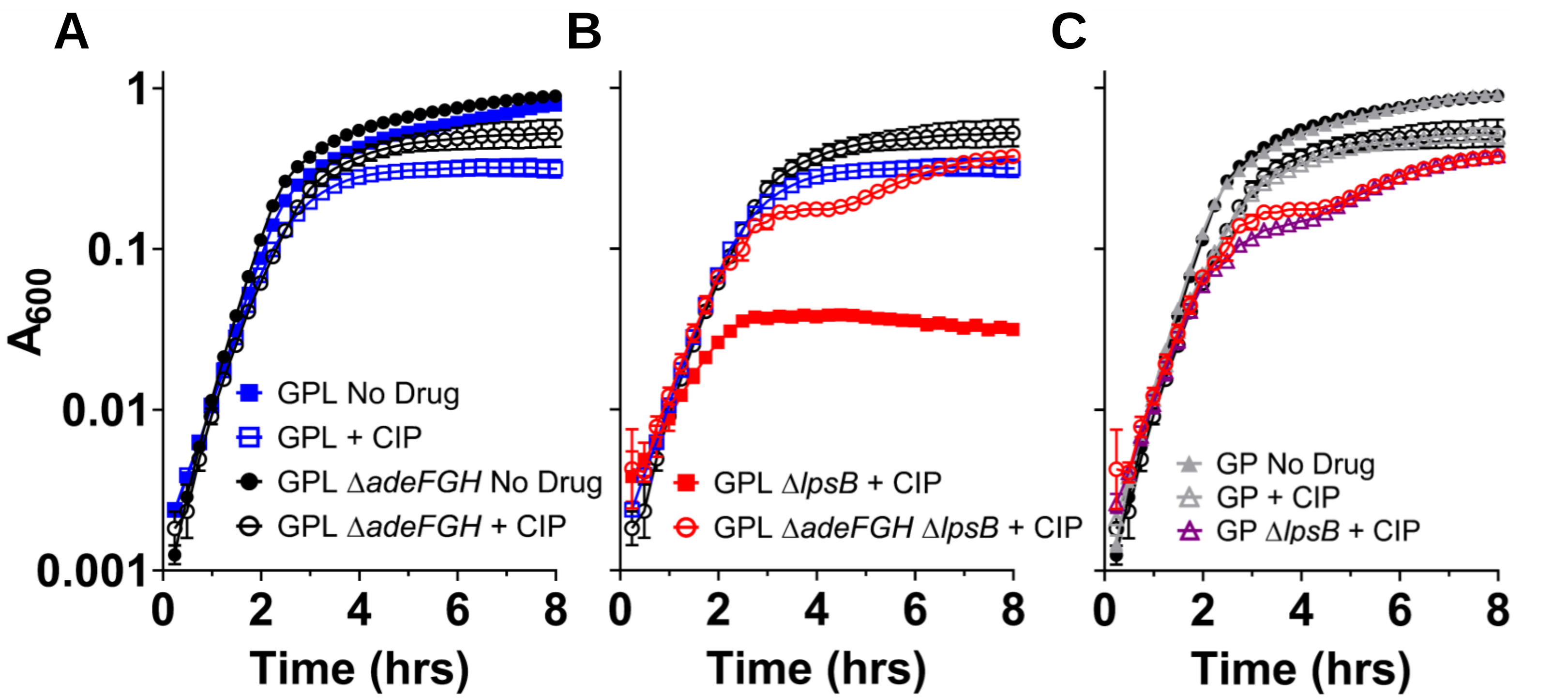
A
B
C
A600
1
0.1
0.01
0.001
0
2
4
6
8
Time (hrs)
GPL No Drug
GPL + CIP
GPL ΔadeFGH No Drug
GPL ΔadeFGH + CIP
GPL ΔlpsB + CIP
GPL ΔadeFGH ΔlpsB + CIP
GP No Drug
GP + CIP
GP ΔlpsB + CIP

Fig. S6

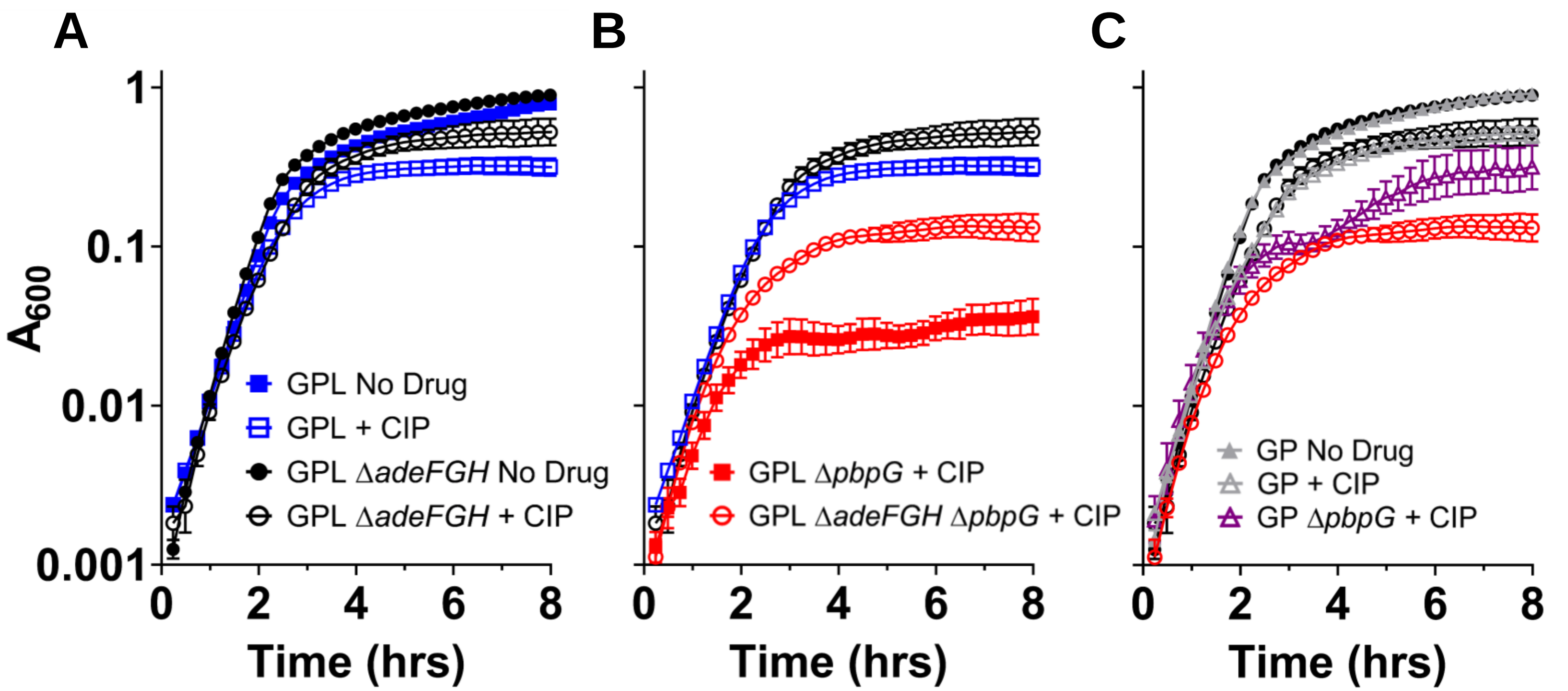

A
B
C
A600
1
0.1
0.01
0.001
0 2 4 6 8
Time (hrs)
GPL No Drug
GPL + CIP
GPL ΔadeFGH No Drug
GPL ΔadeFGH + CIP
GPL ΔpbpG + CIP
GPL ΔadeFGH ΔpbpG + CIP
GP No Drug
GP + CIP
GP ΔpbpG + CIP

Fig. S7

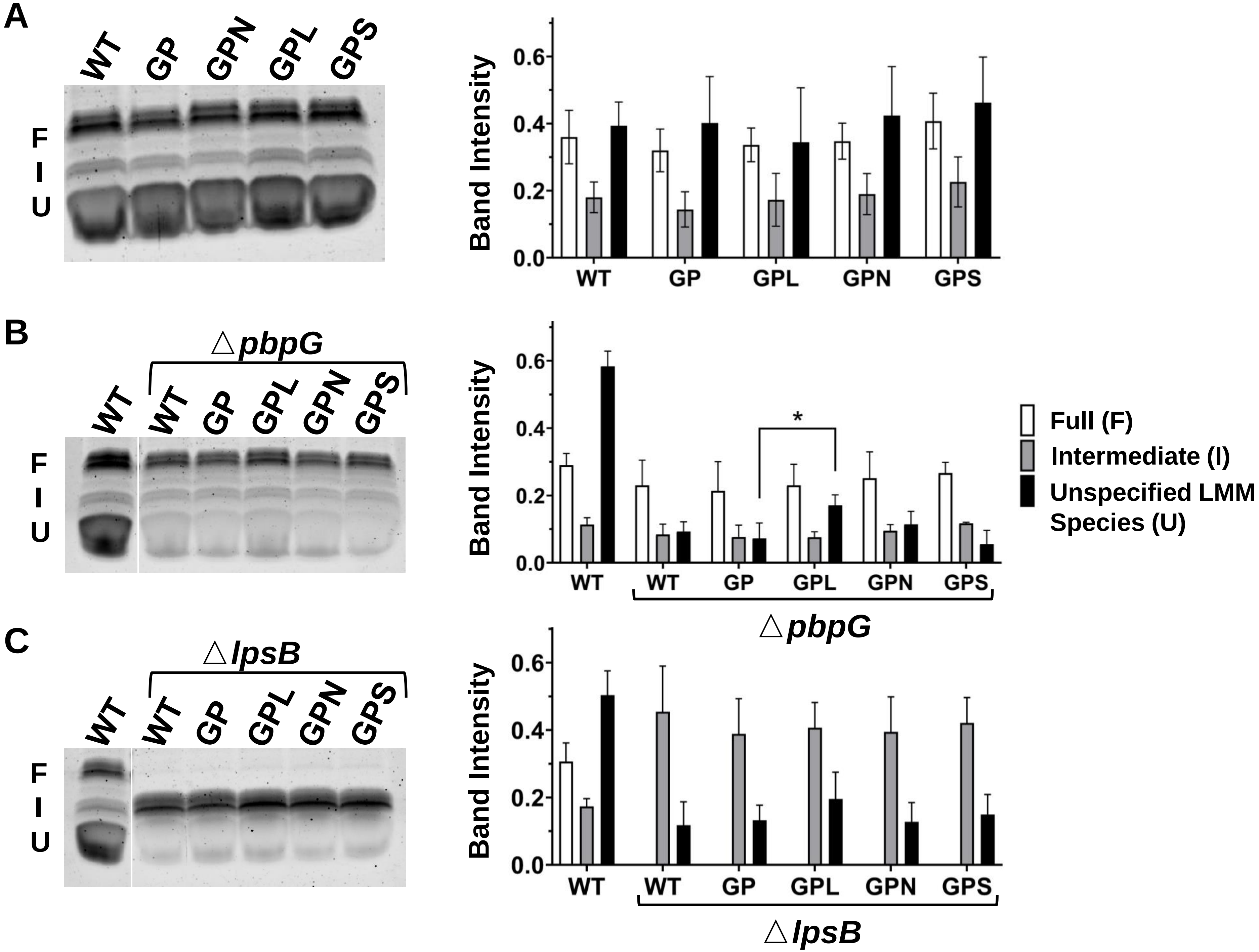
A
WT
GP
GPN
GPL
GPS
F
I
U
Band Intensity
0.6
0.4
0.2
0.0
WT
GP
GPL
GPN
GPS
B
△pbpG
WT
WT
GP
GPL
GPN
GPS
F
I
U
Band Intensity
0.6
0.4
0.2
0.0
*
WT
WT
GP
GPL
GPN
GPS
△pbpG
Full (F)
Intermediate (I)
Unspecified LMM
Species (U)
C
△lpsB
WT
WT
GP
GPL
GPN
GPS
F
I
U
Band Intensity
0.6
0.4
0.2
0.0
WT
WT
GP
GPL
GPN
GPS
△lpsB

Fig. S8

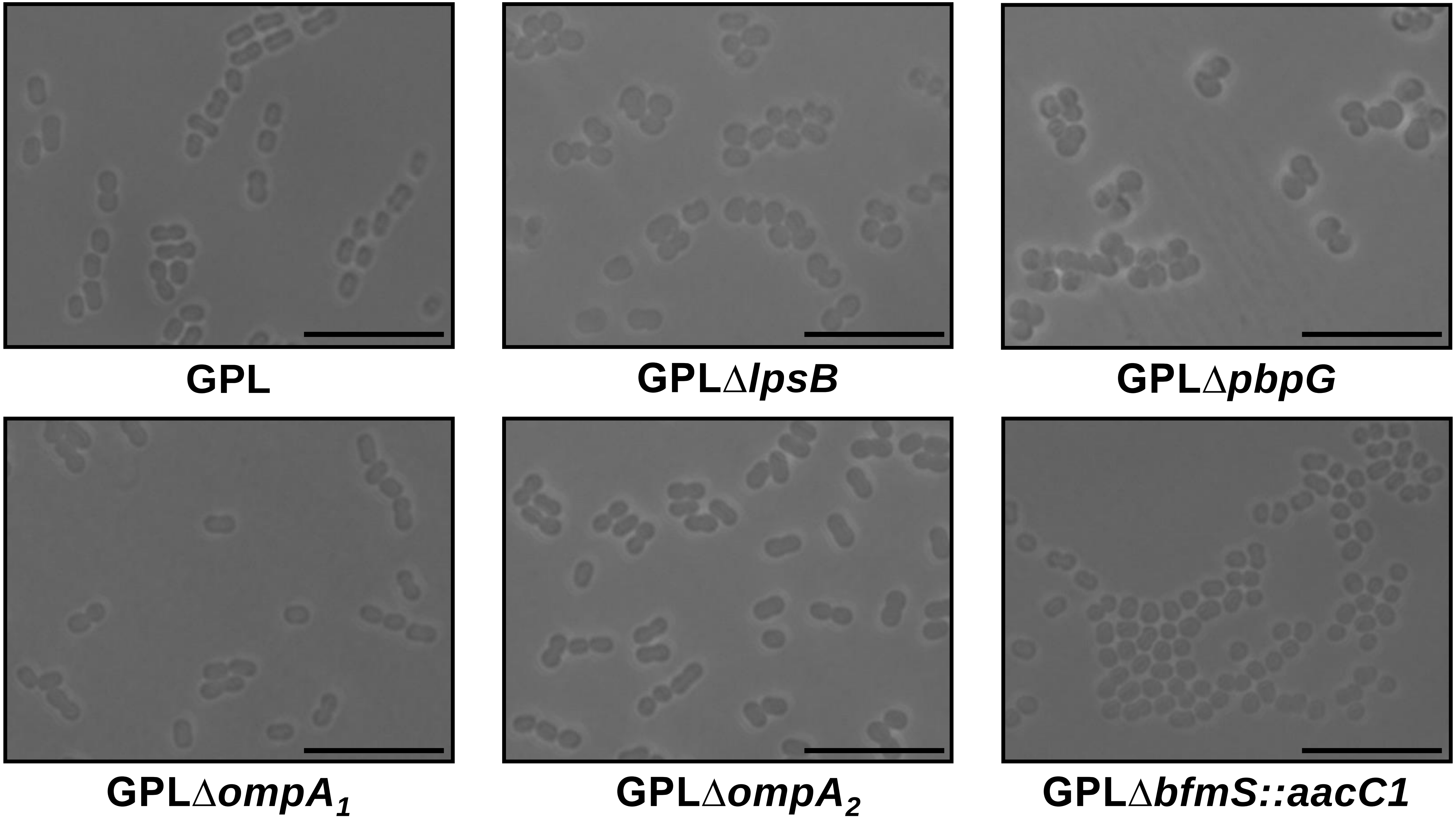
GPL
GPLΔ*lpsB*
GPLΔ*pbpG*
GPLΔ*ompA*$_1$
GPLΔ*ompA*$_2$
GPLΔ*bfmS::aacC1*

Fig. S9

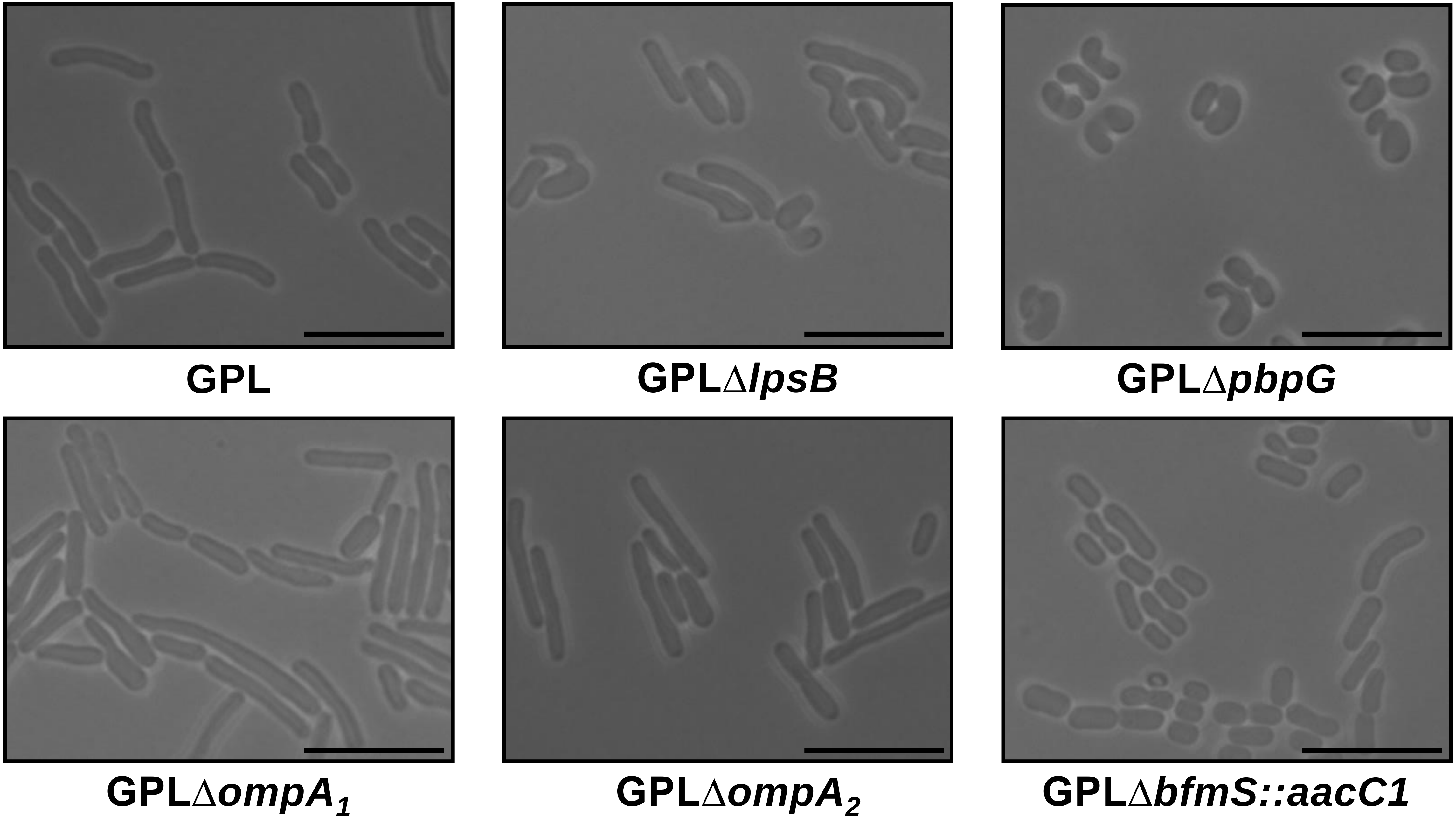
GPL
GPLΔ*lpsB*
GPLΔ*pbpG*
GPLΔ*ompA*$_1$
GPLΔ*ompA*$_2$
GPLΔ*bfmS::aacC1*

Fig. S10

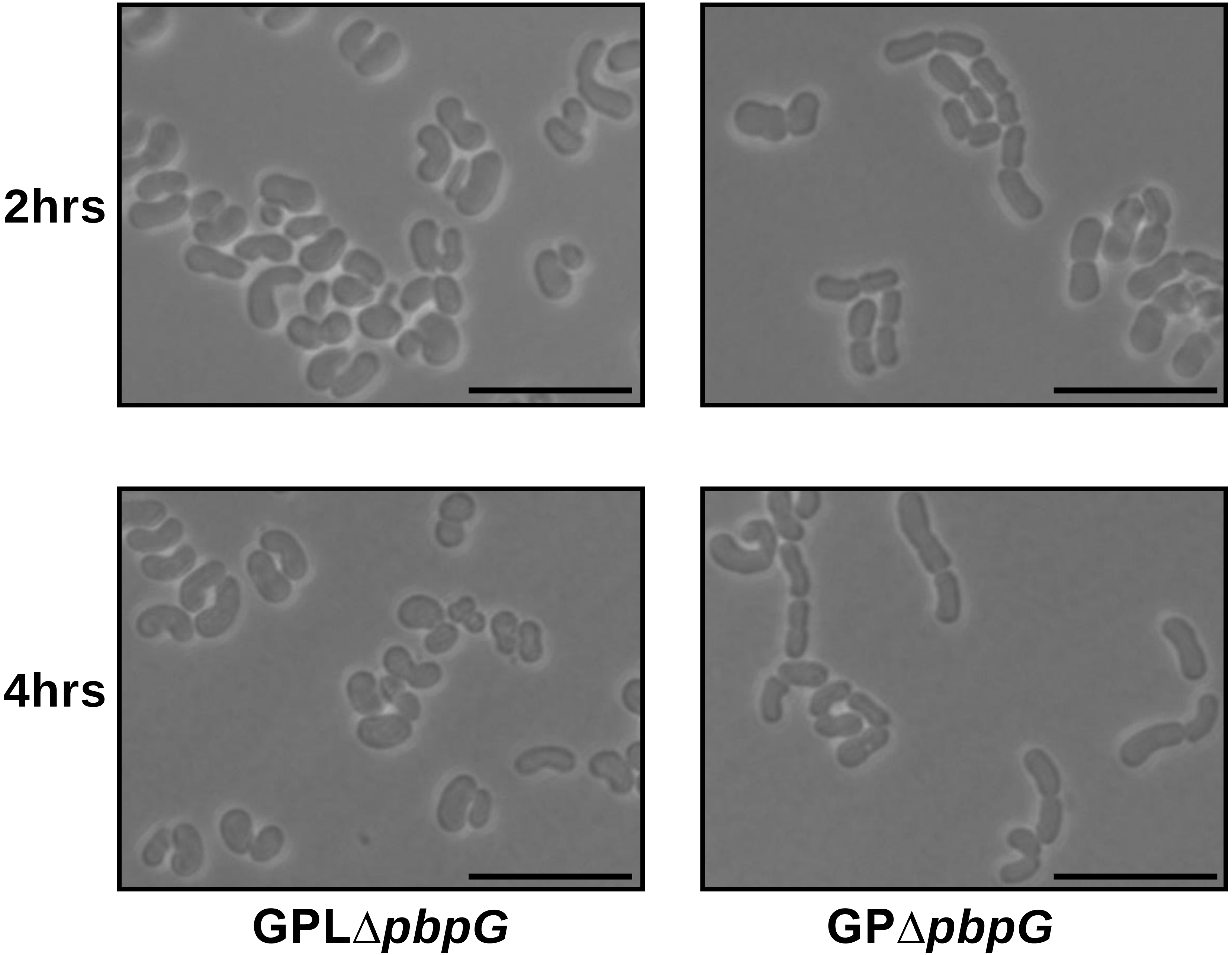

Fig. S11

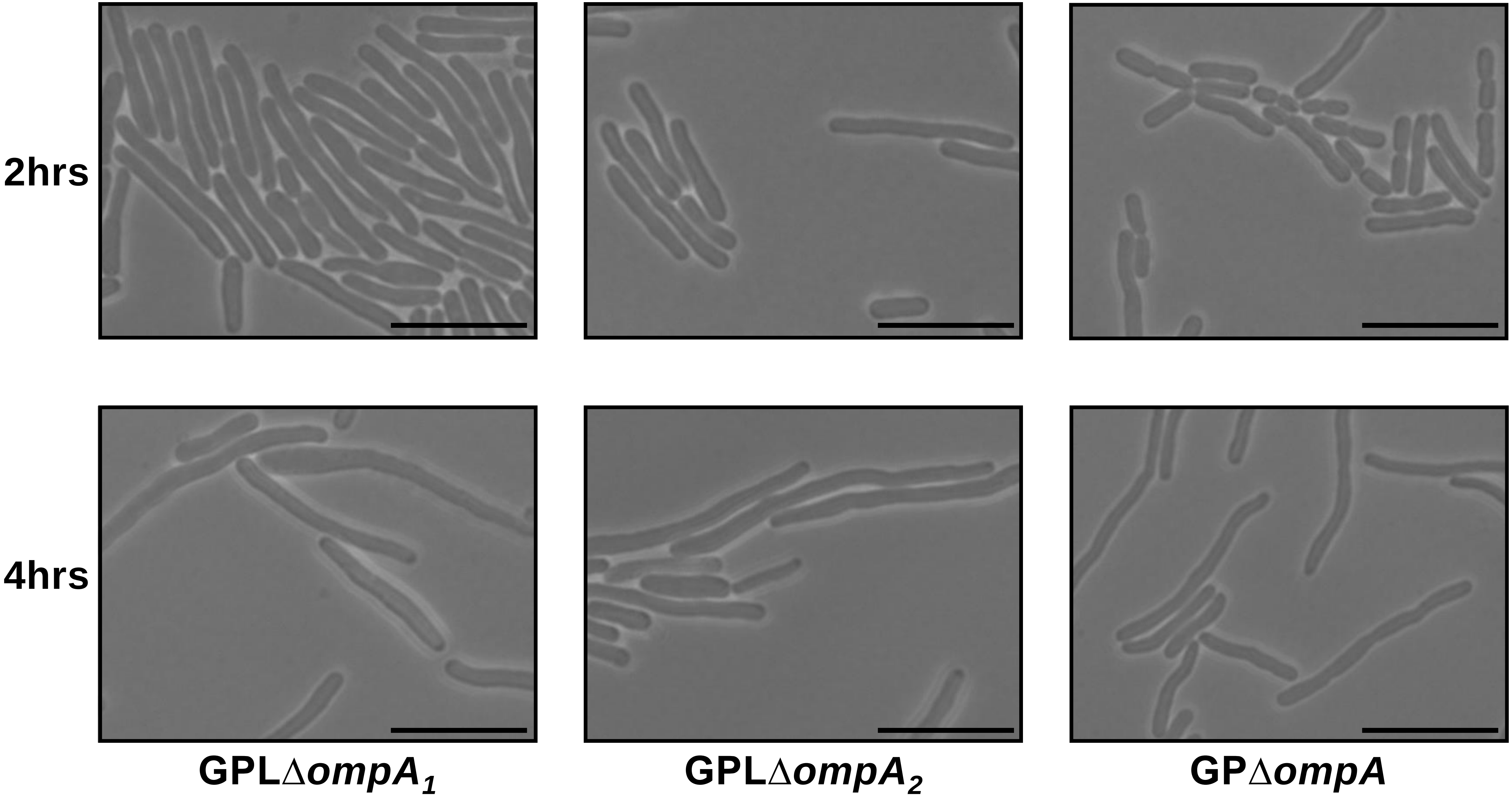

Fig. S12

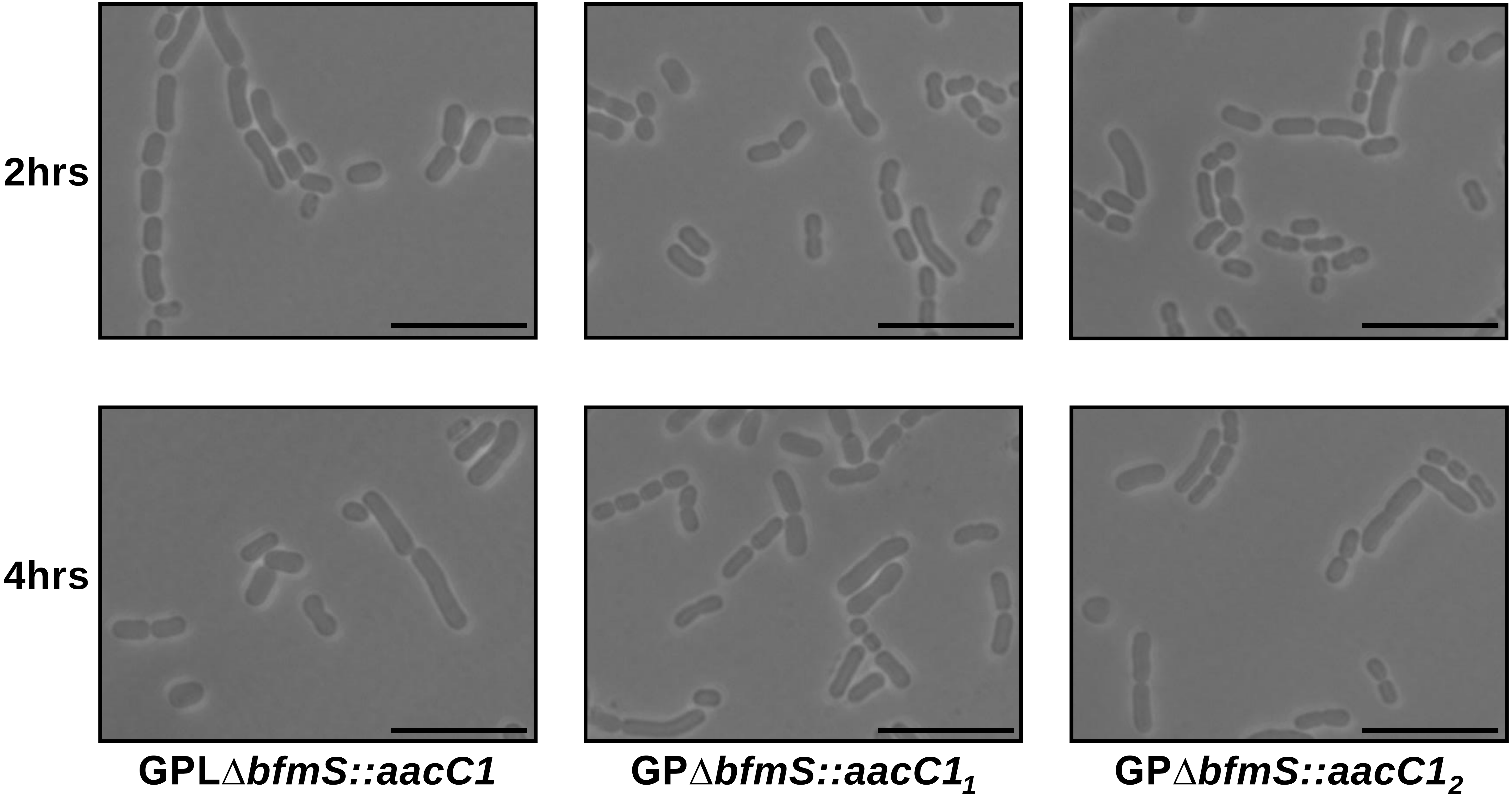

Fig. S13

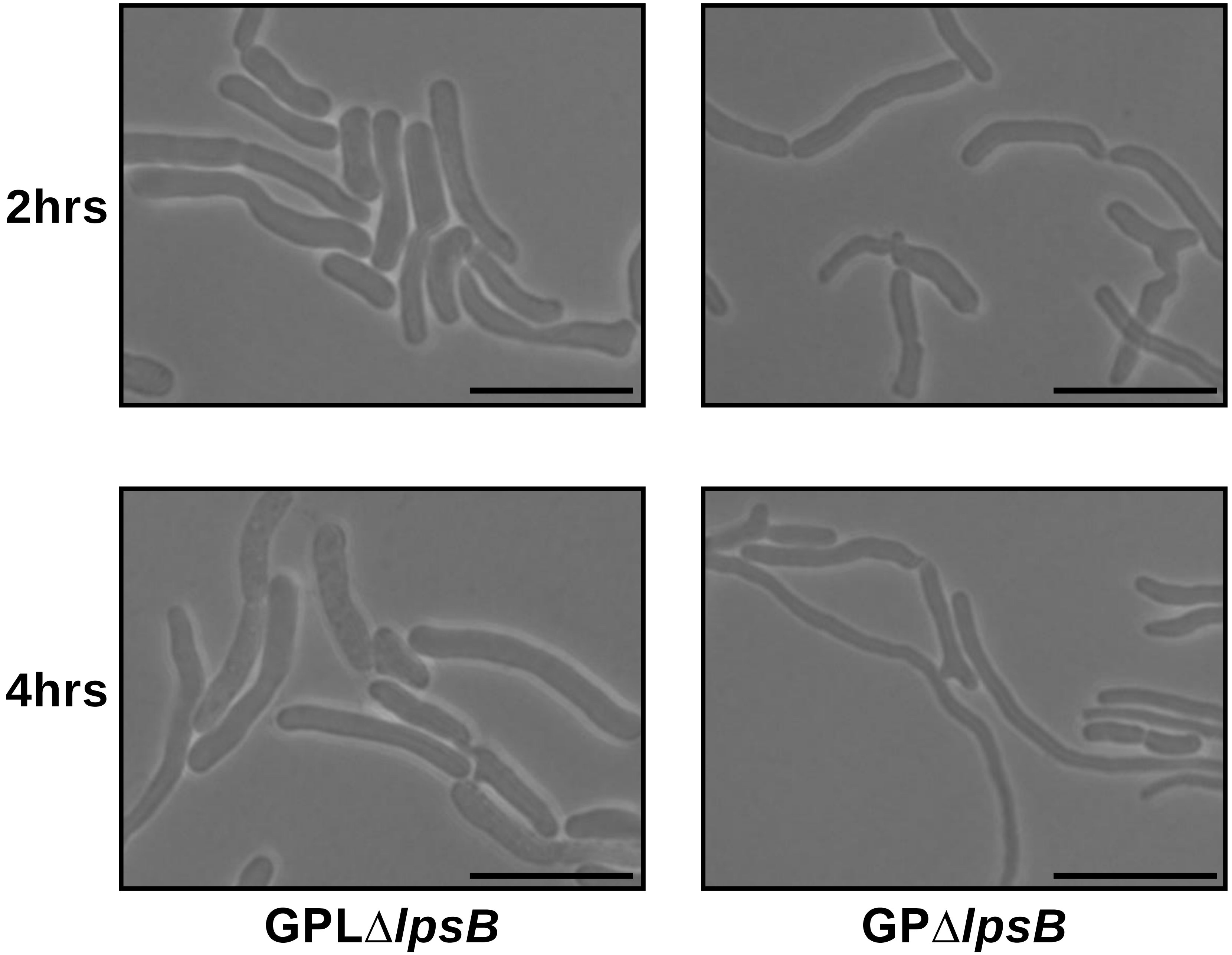

Fig. S14

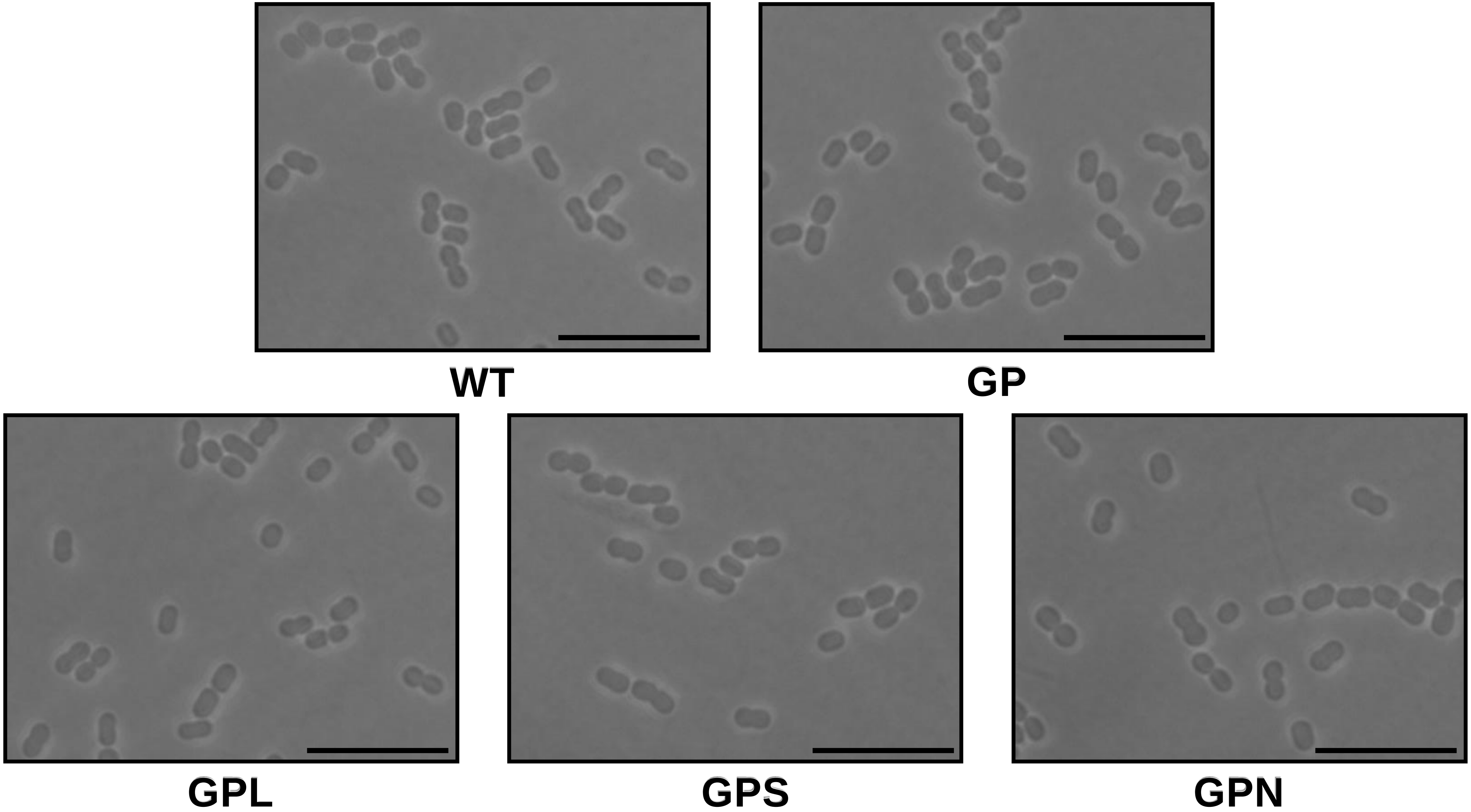

Fig. S15

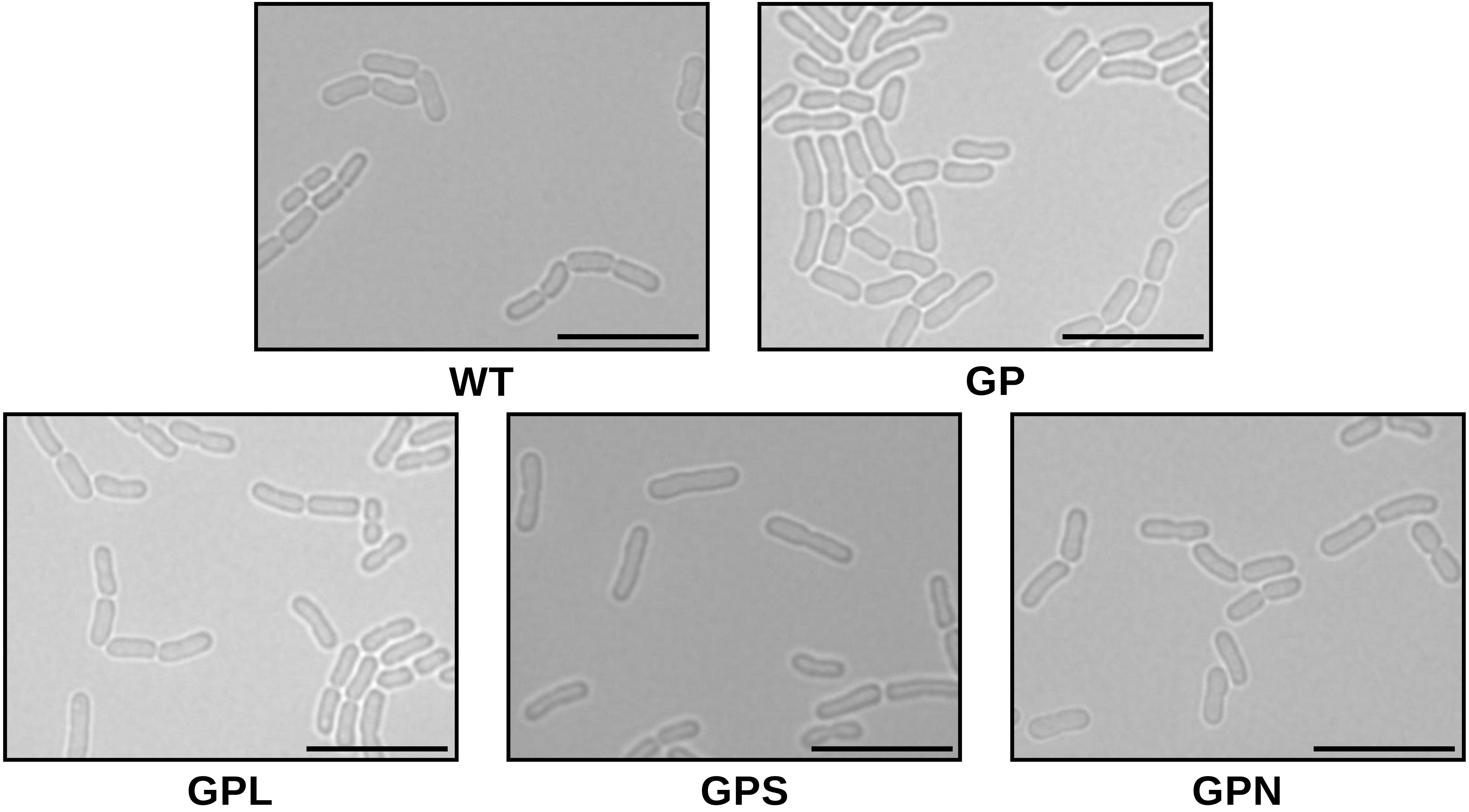

Fig. S16

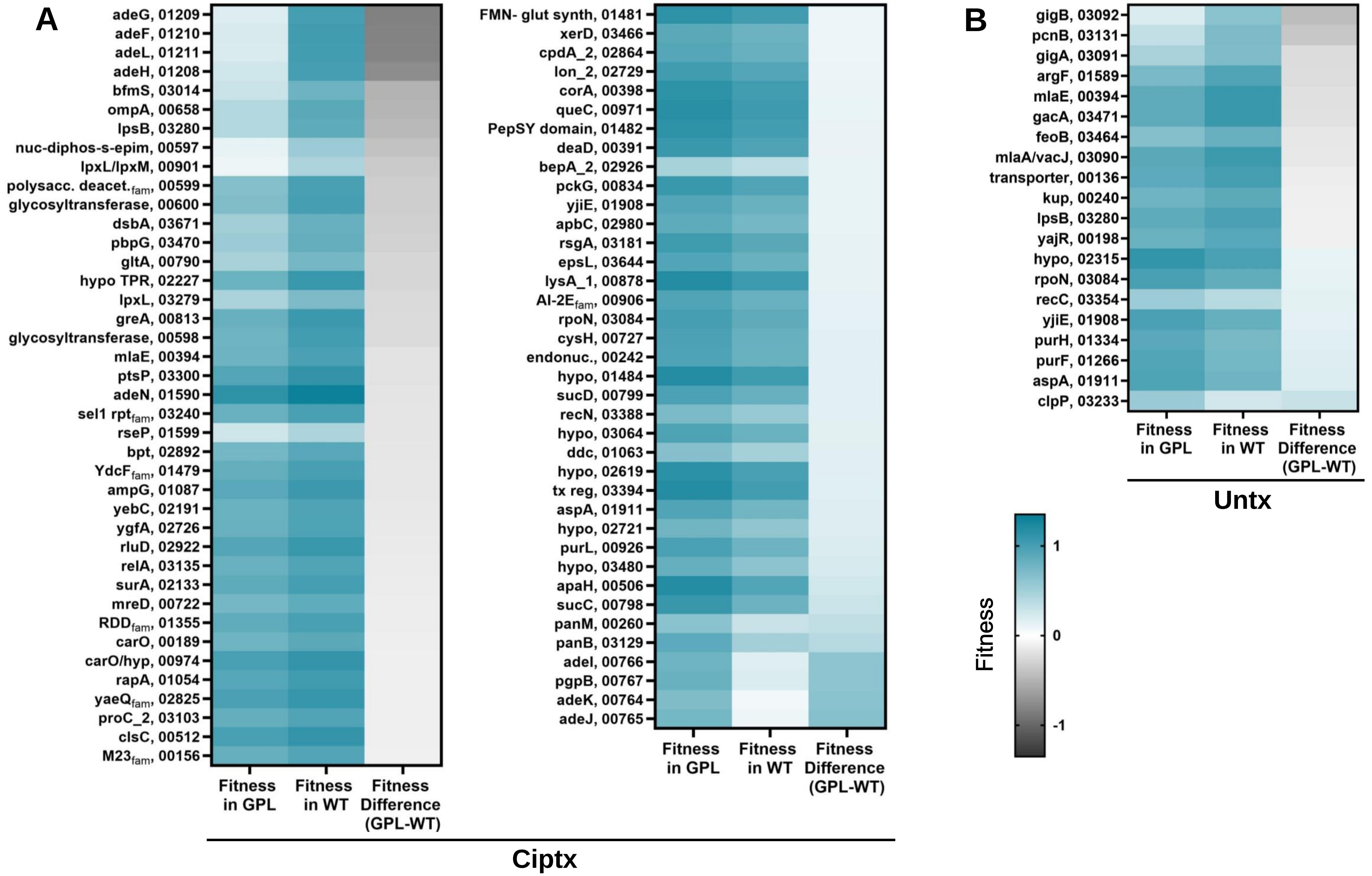
A
adeG, 01209
adeF, 01210
adeL, 01211
adeH, 01208
bfmS, 03014
ompA, 00658
lpsB, 03280
nuc-diphos-s-epim, 00597
lpxL/lpxM, 00901
polysacc. deacet.$_{fam}$, 00599
glycosyltransferase, 00600
dsbA, 03671
pbpG, 03470
gltA, 00790
hypo TPR, 02227
lpxL, 03279
greA, 00813
glycosyltransferase, 00598
mlaE, 00394
ptsP, 03300
adeN, 01590
sel1 rpt$_{fam}$, 03240
rseP, 01599
bpt, 02892
YdcF$_{fam}$, 01479
ampG, 01087
yebC, 02191
ygfA, 02726
rluD, 02922
relA, 03135
surA, 02133
mreD, 00722
RDD$_{fam}$, 01355
carO, 00189
carO/hyp, 00974
rapA, 01054
yaeQ$_{fam}$, 02825
proC_2, 03103
clsC, 00512
M23$_{fam}$, 00156
Fitness in GPL
Fitness in WT
Fitness Difference (GPL-WT)
FMN- glut synth, 01481
xerD, 03466
cpdA_2, 02864
lon_2, 02729
corA, 00398
queC, 00971
PepSY domain, 01482
deaD, 00391
bepA_2, 02926
pckG, 00834
yjiE, 01908
apbC, 02980
rsgA, 03181
epsL, 03644
lysA_1, 00878
AI-2E$_{fam}$, 00906
rpoN, 03084
cysH, 00727
endonuc., 00242
hypo, 01484
sucD, 00799
recN, 03388
hypo, 03064
ddc, 01063
hypo, 02619
tx reg, 03394
aspA, 01911
hypo, 02721
purL, 00926
hypo, 03480
apaH, 00506
sucC, 00798
panM, 00260
panB, 03129
adeI, 00766
pgpB, 00767
adeK, 00764
adeJ, 00765
Fitness in GPL
Fitness in WT
Fitness Difference (GPL-WT)
Ciptx
B
gigB, 03092
pcnB, 03131
gigA, 03091
argF, 01589
mlaE, 00394
gacA, 03471
feoB, 03464
mlaA/vacJ, 03090
transporter, 00136
kup, 00240
lpsB, 03280
yajR, 00198
hypo, 02315
rpoN, 03084
recC, 03354
yjiE, 01908
purH, 01334
purF, 01266
aspA, 01911
clpP, 03233
Fitness in GPL
Fitness in WT
Fitness Difference (GPL-WT)
Untx
Fitness
1
0
-1

Fig. S17

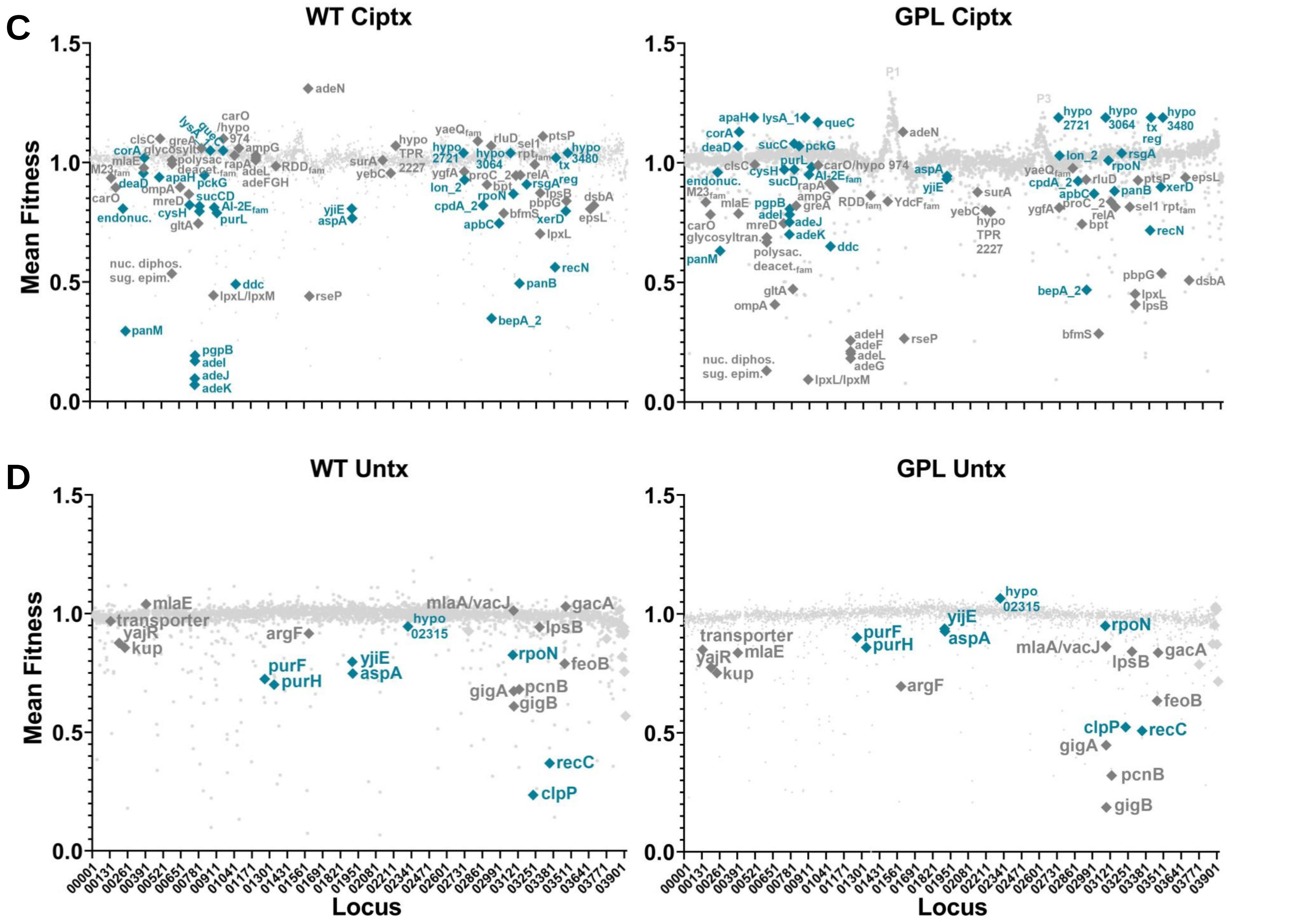
C
WT Ciptx
GPL Ciptx
D
WT Untx
GPL Untx
Mean Fitness
Locus
1.5
1.0
0.5
0.0
00001 00131 00261 00391 00521 00651 00781 00911 01041 01171 01301 01431 01561 01691 01821 01951 02081 02211 02341 02471 02601 02731 02861 02991 03121 03251 03381 03511 03641 03771 03901
adeN
carO /hypo 974
ampG
clsC
greA
glycosylt.
corA
mlaE
M23fam
deaD
apaH
pckG
polysac deacet.fam
rapA
adeL
adeFGH
RDDfam
ompA
mreD
sucCD
cysH
AI-2Efam
purL
carO
endonuc.
gltA
lysA
queC
hypo TPR 2227
surA
yebC
yaeQfam
rluD
sel1
rptfam
ptsP
hypo 2721
hypo 3064
hypo 3480
tx
reg
ygfA
proC_2
relA
bpt
rsgA
lon_2
rpoN
lpsB
dsbA
cpdA_2
pbpG
apbC
bfmS
xerD
epsL
lpxL
yjiE
aspA
nuc. diphos. sug. epim.
ddc
lpxL/lpxM
rseP
recN
panB
bepA_2
panM
pgpB
adeI
adeJ
adeK
P1
P3
lysA_1
sucC
sucD
purL
carO/hypo 974
YdcFfam
glycosyltran.
polysac. deacet.fam
adeH
adeF
adeL
adeG
mlaA/vacJ
gacA
transporter
yajR
kup
argF
hypo 02315
feoB
purF
purH
pcnB
gigA
gigB
recC
clpP

Fig. S17

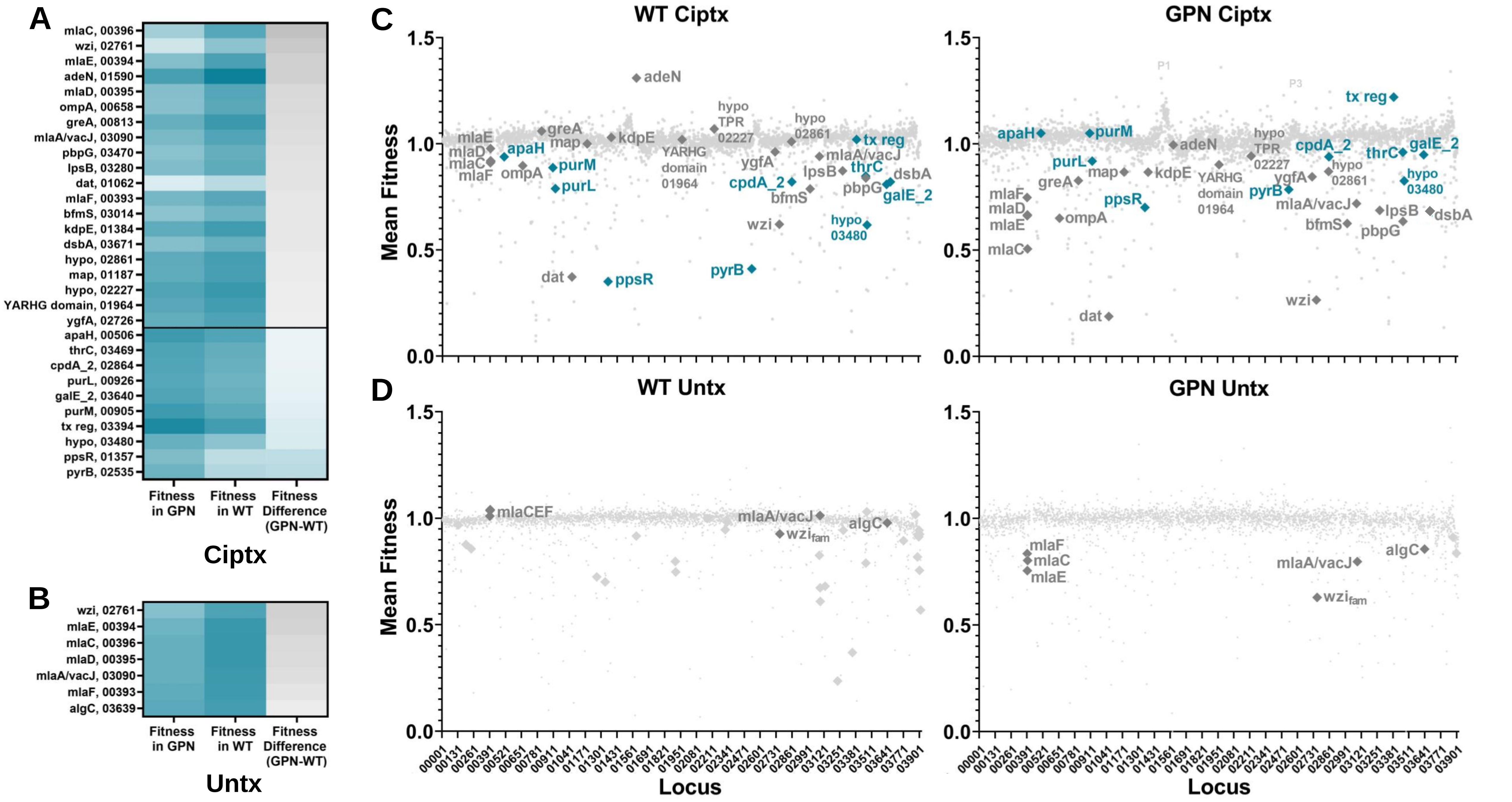
A
mlaC, 00396
wzi, 02761
mlaE, 00394
adeN, 01590
mlaD, 00395
ompA, 00658
greA, 00813
mlaA/vacJ, 03090
pbpG, 03470
lpsB, 03280
dat, 01062
mlaF, 00393
bfmS, 03014
kdpE, 01384
dsbA, 03671
hypo, 02861
map, 01187
hypo, 02227
YARHG domain, 01964
ygfA, 02726
apaH, 00506
thrC, 03469
cpdA_2, 02864
purL, 00926
galE_2, 03640
purM, 00905
tx reg, 03394
hypo, 03480
ppsR, 01357
pyrB, 02535
Fitness in GPN
Fitness in WT
Fitness Difference (GPN-WT)
Ciptx
B
wzi, 02761
mlaE, 00394
mlaC, 00396
mlaD, 00395
mlaA/vacJ, 03090
mlaF, 00393
algC, 03639
Fitness in GPN
Fitness in WT
Fitness Difference (GPN-WT)
Untx
C
WT Ciptx
GPN Ciptx
D
WT Untx
GPN Untx
Mean Fitness
Locus

Fig. S18

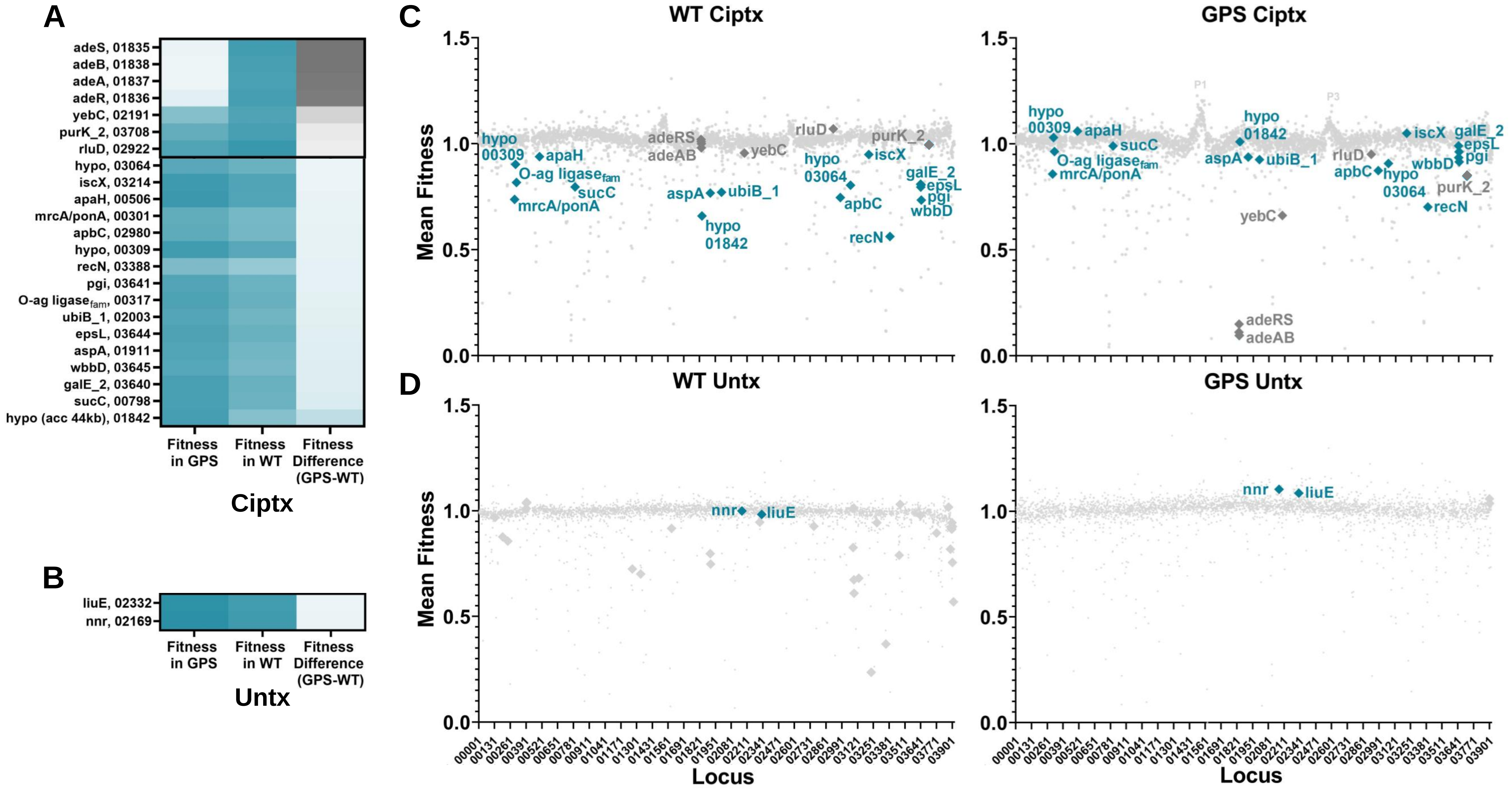
A
adeS, 01835
adeB, 01838
adeA, 01837
adeR, 01836
yebC, 02191
purK_2, 03708
rluD, 02922
hypo, 03064
iscX, 03214
apaH, 00506
mrcA/ponA, 00301
apbC, 02980
hypo, 00309
recN, 03388
pgi, 03641
O-ag ligasefam, 00317
ubiB_1, 02003
epsL, 03644
aspA, 01911
wbbD, 03645
galE_2, 03640
sucC, 00798
hypo (acc 44kb), 01842
Fitness in GPS
Fitness in WT
Fitness Difference (GPS-WT)
Ciptx
B
liuE, 02332
nnr, 02169
Fitness in GPS
Fitness in WT
Fitness Difference (GPS-WT)
Untx
C
WT Ciptx
GPS Ciptx
Mean Fitness
D
WT Untx
GPS Untx
Mean Fitness
Locus

Fig. S19

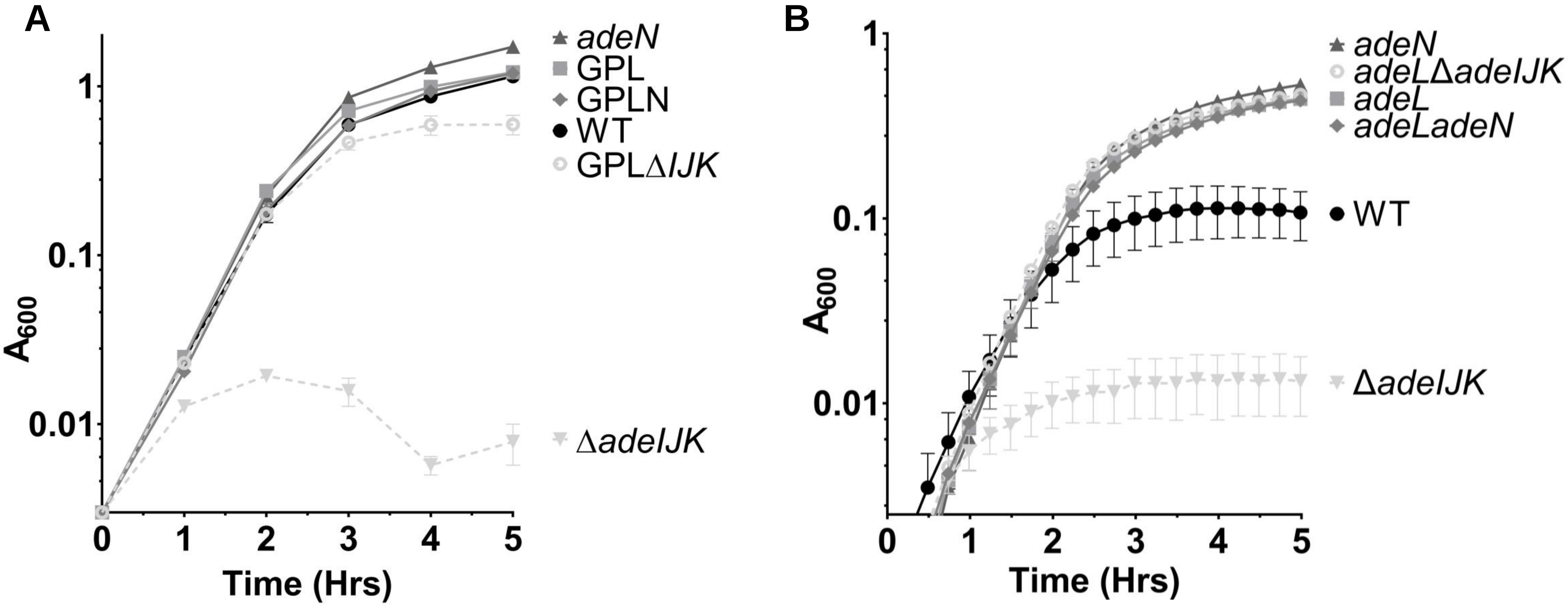

A
adeN
GPL
GPLN
WT
GPLΔIJK
ΔadeIJK
A600
1
0.1
0.01
0
1
2
3
4
5
Time (Hrs)
B
adeN
adeLΔadeIJK
adeL
adeLadeN
WT
ΔadeIJK
A600
1
0.1
0.01
0
1
2
3
4
5
Time (Hrs)

Fig. S20

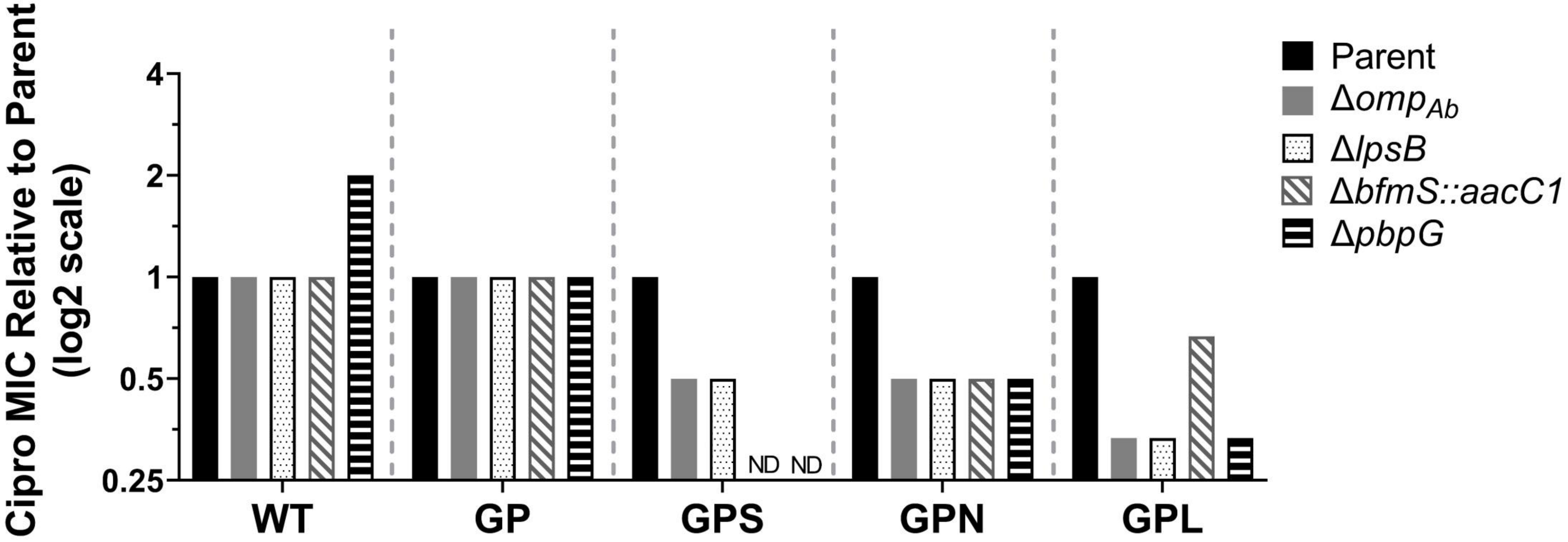

Cipro MIC Relative to Parent (log2 scale)
4
2
1
0.5
0.25
ND
ND
WT
GP
GPS
GPN
GPL
Parent
Δomp_{Ab}
Δ*lpsB*
Δ*bfmS::aacC1*
Δ*pbpG*

Fig. S21
